# Early Response to Dehydration Six-Like Transporter Family: Early Origin in Streptophytes and Evolution in Land Plants

**DOI:** 10.3389/fpls.2021.681929

**Published:** 2021-09-06

**Authors:** Lucie Slawinski, Abir Israel, Caroline Paillot, Florence Thibault, Richard Cordaux, Rossitza Atanassova, Fabienne Dédaldéchamp, Maryse Laloi

**Affiliations:** Laboratoire Ecologie et Biologie des Interactions, UMR Centre National de la Recherche Scientifique 7267, Université de Poitiers, Poitiers, France

**Keywords:** sugar transporters, monosaccharides carriers, ERD6-like, ESL, evolution, gene duplication, *Arabidopsis*

## Abstract

Carbon management by plants involves the activity of many sugar transporters, which play roles in sugar subcellular partitioning and reallocation at the whole organism scale. Among these transporters, the early response to dehydration six-like (ESL) monosaccharide transporters (MSTs) are still poorly characterized although they represent one of the largest sugar transporter subfamilies. In this study, we used an evolutionary genomic approach to infer the evolutionary history of this multigenic family. No ESL could be identified in the genomes of rhodophytes, chlorophytes, and the brown algae *Ectocarpus siliculosus*, whereas one ESL was identified in the genome of *Klebsormidium nitens* providing evidence for the early emergence of these transporters in Streptophytes. A phylogenetic analysis using the 519 putative ESL proteins identified in the genomes of 47 Embryophyta species and being representative of the plant kingdom has revealed that ESL protein sequences can be divided into three major groups. The first and second groups originated in the common ancestor of all spermaphytes [ζ: 340 million years ago (MYA)] and of angiosperms (ε: 170–235 MYA), respectively, and the third group originated before the divergence of rosids and asterids (γ/1R: 117 MYA). In some eudicots (Vitales, Malpighiales, Myrtales, Sapindales, Brassicales, Malvales, and Solanales), the ESL family presents remarkable expansions of gene copies associated with tandem duplications. The analysis of non-synonymous and synonymous substitutions for the dN/dS ratio of the *ESL* copies of the genus *Arabidopsis* has revealed that *ESL* genes are evolved under a purifying selection even though the progressive increase of dN/dS ratios in the three groups suggests subdiversification phenomena. To further explore the possible acquisition of novel functions by ESL MSTs, we identified the gene structure and promoter *cis*-acting elements for *Arabidopsis thaliana ESL* genes. The expression profiling of *Arabidopsis ESL* unraveled some gene copies that are almost constitutively expressed, whereas other gene copies display organ-preferential expression patterns. This study provides an evolving framework to better understand the roles of ESL transporters in plant development and response to environmental constraints.

## Introduction

In higher plants, sugars display numerous important roles. They constitute the sources of cellular energy and carbon skeletons necessary for different biosynthetic pathways such as starch and cellulose biosynthesis. Acting as compatible osmolytes, they can prevent cellular damages caused by various abiotic stresses. Sugars also represent important signal molecules. They are distributed through the plant *via* sugar transporters, which are involved not only in the long-distance transport of sugars via the loading and unloading of the conducting complex but also in the sugar allocation from source and sink organs. Sugar carriers belong to a major facilitator superfamily (MFS), including disaccharide transporters [sucrose transporter (SUC)/suc transporter (SUT)] (Sauer, 2007; Reinders et al., 2012) and monosaccharide transporters (MSTs) (Büttner, 2007), which are characterized by 12 transmembrane domains, and also belong to the sugar will be eventually exported transporter (SWEET) family (Chen et al., 2010) characterized by only seven transmembrane domains. The MST family can be divided into seven subfamilies, and the early response to dehydration six-like (ESL) subfamily is one among the subfamilies. The ESL family is in many species the largest subfamily, encompassing 19 members in *Arabidopsis* (Büttner, 2007) and 22 in *Vitis vinifera* (Afoufa-Bastien et al., 2010). The lineage *Brassica* presents a very large ESL subfamily, with 24 and 30 ESL copies between *B. rapa, B. oleracea*, and the allotetraploid *B. napus* (A and C subgenomes) (Zhang et al., 2020). In Fabaceae, the ESL family is as small as Medicago (*Medicago truncatula;* comprising *10 MtESL*) and pea (*Pisum sativum*; comprising 9 PsESL) (Doidy et al., 2019). Similarly, the genome of *Malus domestica* (Wei et al., 2014), *Solanum lycopersicum* (Reuscher et al., 2014), *Ricinus communis* (Mao et al., 2017), and *Oryza sativa* (Deng et al., 2019) contains 11, 10, 9, and 6 *ESL* genes, respectively. The genome of root parasitic angiosperm weeds, such as *Striga hermonthica, Triphysaria versicolor, Mimulus guttatus*, and *Phelipanche aegyptiaca*, contains 8, 6, 6, and 5 *ESL* genes, respectively (Misra et al., 2019). By searching the EST databases of various species representing all terrestrial plants, Johnson et al. (2006) could detect one ESL gene in the genomes of *Selaginella* and *P. patens*, four in *Pinus taeda* and *Zea mays*. Lalonde and Frommer (2012) also identified one *ESL* gene in the genome of *Selaginell*a but none in *P. patens* genome. These findings demonstrate that ESL genes are also present in early diverging embryophyte lineages. The comparison of *Arabidopsis* and rice revealed that in rice, the sugar transport protein (STP) and polyol transporter (PLT) subfamilies were greatly expanded, whereas the ESL subfamily was much larger in *Arabidopsis* (Johnson and Thomas, 2007). This difference might be due to tandem duplication events in their evolutionary histories, and it has been proposed that the expansion of the ESL subfamily occurs via two segmental duplications and six tandem duplications (Johnson et al., 2006). Finally, in Grapevine (*V. vinifera*), 14 VvESL open reading frames were found to be located in tandem on chromosome 14 (Afoufa-Bastien et al., 2010). Altogether, these results put into question the biological relevance of so many duplicated *ESL* genes in some plant genomes.

Despite a high number of genes, only a few *ESL* genes have been studied until now, and their biological functions remain largely unknown. The first ESL gene, named as ERD6 (*At1g08930*), has been identified by using a differential screening of the complementary DNA (cDNA) library from *Arabidopsis thaliana* plants exposed to dehydration stress (Kiyosue et al., 1994). *AtERD6* expression is upregulated by drought (Kiyosue et al., 1998; Seki et al., 2002; Yamada et al., 2010) and low temperature (Kiyosue et al., 1998) and is repressed in leaves by high salinity and ABA (Yamada et al., 2010). AtESL1 (for ERD six-like 1—*At1g08920*) is a low affinity facilitator, which is able to transport different hexoses (glucose, fructose, galactose, mannose, and xylose) across the tonoplast. Its expression is highly upregulated by high salinity and ABA in roots and slightly induced by drought (Yamada et al., 2010). *AtSFP1* and *AtSFP2* (sugar porter family proteins 1 and 2—*At5g27350* and *At5g27360*) are two tandem duplicated genes located on chromosome 5. These two genes displayed different expression patterns during leaf development. *AtSFP1* was detected in seedlings 9 days after germination but not detected in mature plant organs (leaves, flowers, flower buds, stem, and roots), whereas *AtSFP2* was expressed in all tested plant organs and in seedlings. Further analysis showed that only *SFP1* is induced during leaf senescence but the expression of *AtSFP2* remains stable during this process (Quirino et al., 2001). AtZIF2 (a zinc-induced facilitator—At2g48020) is a tonoplastic transporter, which is expressed in different organs, mainly in the roots, to promote the sequestration of zinc into the vacuole (Remy et al., 2014). AtERDL6 (Early Responsive to Dehydration-Like six - At1g75220) is a tonoplastic H^+g^/glucose symporter involved in the export of glucose from the vacuole under the conditions that require the mobilization of vacuolar carbohydrate reserves. The expression of *AtERDL6* was upregulated under the conditions of darkness, heat stress, and wounded leaves and was downregulated under cold stress and exogen glucose supply, allowing its accumulation in the vacuole (Poschet et al., 2011). Furthermore, *atesl1.2* mutant showed an increase in the vacuolar glucose content (more than 90%) compared to the wild type (86%) (Poschet et al., 2011), whereas *Arabidopsis* lines overexpressing *AtERDL6* had lower glucose levels than wild-type plants (Klemens et al., 2014). Only three ESL genes from other species has been characterized. The apple MdERDL6-1, a homolog of AtERDL6 in *Arabidopsis*, was recently identified. It is highly expressed in fruits; its encoded protein seems to be localized at the tonoplast and acts as an H+/glucose symporter with a low affinity for glucose. In contrary to AtERDL6, the overexpression of MdERDL6-1 leads to an increase rather than a decrease in sugar levels in apple and the leaves and fruits of tomato (Zhu et al., 2021). In Pineapple (*Ananas comosus*), the screening of a leaf cDNA library using a degenerated PCR led to the identification of AcMST1, an ESL localized in the tonoplast (Antony et al., 2007). Breia et al. (2020) showed that the grapevine *VvERD6l13* gene was induced in fruits upon infection by necrotrophic or biotrophic pathogens such as *Botrytis cinerea* and *Erysiphe necator*. VvERD6l13 is the first ESL described to be localized in the plasma membrane. Even more surprisingly, using yeast as a heterologous system, VvERD6l13 has been characterized as a low affinity H^+^/sucrose symporter (Breia et al., 2020). Moreover, Desrut et al. (2020) showed that six *AtESL* genes were differentially regulated in the presence of plant growth-promoting rhizobacteria (PGPR). Indeed, *ERD6-like7, ERD6-like12*, and *ERD6-like16*, as well as *ERD6-like18*, were downregulated in shoots and roots, respectively, whereas *ERD6-like13* and *ERD6-like15* were upregulated in roots in response to PGPR (Desrut et al., 2020). Taken together, the above-cited studies emphasize the structural and functional diversity of ESL transporters. The transporters, which are localized on the tonoplast or plasma membrane, can transport not only monosaccharides but also sucrose and might be involved in many plant responses to abiotic and biotic environmental cues. The biological relevance of so many duplicated *ESL* genes in several plant genomes prompted us to investigate the evolutionary history of the ESL subfamily. For this, we performed a phylogenetic analysis using the ESL proteins identified in 47 embryophyte proteomes, and identified tandem duplicates copies, gene structures and ESL protein motifs. We analyzed the selection regime experienced by the Brassicaceae family, studied, for *A. thaliana*, the gene expression pattern, and searched the putative *cis*-regulatory motifs in *AtESL* promoters.

## Materials and Methods

### Plant Materials and Growth Conditions

*Arabidopsis thaliana* plants (ecotype Columbia: Col-0) were grown in Arasystem^TM^ (BETATECH bvba, Ghent, Belgium) containing an autoclaved mix of compost/vermiculite (3/1) into a growth chamber under a 10-h light (22°C)/14 h dark (18°C) photoperiod, with 50% (light) to 90% (dark) relative humidity. Fully expanded leaves, roots, flower buds, flowers, and siliques were used for sampling. The roots were separated from soil by multiple careful washings with water before sampling.

### ERD6-Like Sequence Identification

Previously identified ESL proteins of *A. thaliana, Brachypodium distachyon, Cucumis melo, Musa acuminata, O. sativa, Populus trichocarpa, Solanum lycopericum, V. vinifera*, and *Z. mays* were collected from the Aramemnon online database (http://aramemnon.uni-koeln.de). The ESL sequences from other species were identified through the protein basic local alignment search tool (BLASTp) analysis (Altschul, 1997) using the 18 full-length *V. vinifera* ESL protein sequences as queries (Afoufa-Bastien et al., 2010) and an *E*-value of 10^−60^ against the online server Phytozome v10.0 (https://phytozome.jgi.doe.gov/pz/portal.html) with the exceptions of *P. taeda* (http://congenie.org), *Galdieria sulphuraria, Chondrus crispus, Pyropia yezoensis, Chlorella vulgaris, Phoenix dactylifera, Nelumbo nucifera, Cicer arietinum, Genlisea aurea, Ectocarpus siliculosus* (https://www.ncbi.nlm.nih.gov), *Lotus japonicus* (http://www.kazusa.or.jp/lotus/), and *Klebsormidium nitens* (https://phycocosm.jgi.doe.gov/Klenit1/Klenit1.home.html). Only full-length sequences were collected, and the potential isoform sequences were eliminated from the study. The species and the identified sequences are summarized in Supplementary Table 1.

### Phylogenetic Analysis

Alignments of the identified ESL protein sequences were performed by using the BioEdit software (https://bioedit.software.informer.com) and the ClustalW method. A phylogenetic analysis was performed by using the MEGA v.6.0 software (Tamura et al., 2013) and the Maximum Likelihood method [the phylogeny software based on the maximum-likelihood principle (PhyML)] with 1,000 bootstrap replicates. The prior alignment performed by MEGA v.6.0 followed the Jones–Taylor–Thornton (JTT) amino acid model. The sucrose transporter (SUC) *A. thaliana* sucrose carrier 2 (AtSUC2) protein sequence and one referent of each monosaccharide transporter (MST) subfamily [*A. thaliana* PLT 5 (AtPLT5), *A. thaliana* inositol transporter 1 (AtINT1), *A. thaliana* STP 13 (AtSTP13), *A. thaliana* vacuolar glucose transporter 1 (AtVGT1), *A. thaliana* tonoplastic monosaccharide transporter (AtTMT1), and *A. thaliana* plastidic glucose transporter 1 (AtpGlt)] were used as an outgroup to root the phylogenetic tree. From the inference of a phylogenetic tree, all dates, periods, and evolutionary data used to reconstruct the evolutionary history of the ESL family in land plants were obtained from the Time Tree of Life (http://www.timetree.org) (Hedges et al., 2006; Kumar et al., 2017).

### Estimation of Selection Pressures

*Early response to dehydration six-like* coding sequences (CDSs) from *A. thaliana, Arabidopsis lyrata, Arabidopsis halleri*, and *Capsella rubella* were aligned by MEGA 6.0 using the neighbor-joining method, 1,000 bootstrap, and the JTT amino acid substitution model. This led to the definition of the 18 monophyletic groups of *ESL* sequences, each containing at least 1 *A. thaliana ESL* sequence as well as a sequence of *C. rubella* (when possible: 16 of the 18 groups). For each group, a second alignment was performed by using the BioEdit free software (https://bioedit.software.informer.com) and ClustalW method and was manually realigned to keep the same open reading frame for all the aligned CDS. In total, four sequences (Ah04425s02, Cr10000845, Cr10015811, and Cr10021344) were removed from the study due to non-possiblity of aligning the sequences correctly. The dN/dS (KaKs) ratio of *ESL* sequences was calculated by the Datamonkey online server (http://www.datamonkey.org), using the GA-branch method (Kosakovsky Pond and Frost, 2005; Delport et al., 2010) following the HKY85 model. The user tree set was defined as (((Ah; Al), At), Cr), which reflected the evolution of these four species.

### Gene Structure and Protein Motifs Determination

*Early response to dehydration six-like* gene structures were determined by using the GSDS v.2.0 online server (Gen Structure Display Server: http://gsds.cbi.pku.edu.cn/) developed by Beijing University's Bio-Informatics Center (Hu et al., 2015). Gene structures were determined *via* the alignment of genomic sequence and CDSs. Each sequence was represented with all exons in the same scale and the same size for all introns. *L. japonicus* sequences were not included.

The conserved motifs of *ESL* and other sugar transporters were analyzed by using multiple EM for motif elicitation (MEME) suite 5.3.3 program (https://meme-suite.org/meme/tools/meme) with the following parameters: optimum width, 3–60; the maximum number of motifs 50, and an *E*-value of 10^−60^.

### Promoter Analysis

About 1,000-bp-long promoter sequences of 19 *AtESL* genes were retrieved from The *Arabidopsis* Information Resource (TAIR) database. These sequences were then scanned for the presence of *cis*-regulatory elements by using the plant cis-acting regulatory DNA element (PLACE) database (https://www.dna.affrc.go.jp/PLACE/?action=newplace) (Higo et al., 1999).

### RNA Extraction and Real-Time Quantitative PCR Analysis

Total RNA was extracted from the frozen ground *A. thaliana* tissues according to Kay et al. (1987). RNA quantity and quality were verified by using a Microdrop (Thermo Fischer, Waltham, MA, USA) and an agarose gel. Total RNA was treated with DNAse I (Sigma-Aldrich, St. Louis, MO, USA), and the reverse transcription was performed by using M-MLV reverse transcriptase (Promega, Madison, WI, USA). Real-time quantitative PCR (RT-qPCR) was carried out by using the GoTaq qPCR Master Mix (Promega, Madison, WI, USA) with a Mastercycler *realplex*^2^ instrument (Eppendorf, Hamburg, Germany). The results of the target gene expression were normalized with the average Ct value of the reference gene, *AtPP2a* (*At1g13320*) (Czechowski et al., 2005). The results were expressed as the relative gene expression according to the 2^−ΔCt^ method. Primers were designed by using Oligo 7 and tested for specificity and efficiency (≥90%). The primer sequences used in this study are listed in Supplementary Table 2.

### Statistical Analyses

Statistical analyses were performed by using XLStats 2011 (Addinsoft, Paris, France).

## Results

### ESL Transporters Emerged With Streptophyta and Diverged Into Three Major Groups in Terrestrial Plants

**Figure 1:**
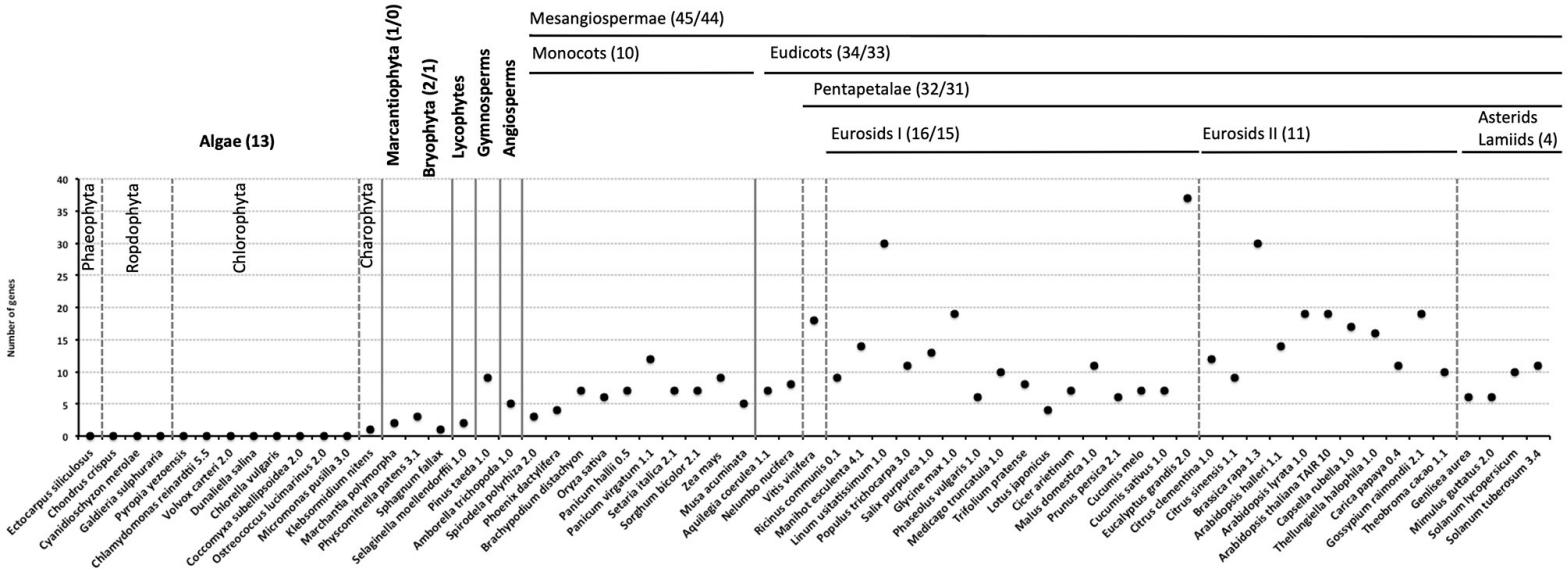
The number of early response to dehydration six-like (ESL) proteins identified in each species. The classification of the species is indicated at the top of the graph. Numbers in bracket indicate the total sequence number/the number of sequences included in a phylogenetic analysis.

To study the evolution of ESL transporters, we performed the protein BLAST (BLASTp) analysis using 18 *V. vinifera* ESL protein sequences as queries against 63 proteomes from photosynthetic organisms representing the main groups of algae and the plant kingdom. BLASTp analysis of the genomes of 13 algae, including stramenopiles (*E. siliculosus*), rhodophytes (*C. crispus, Cyanidioschyzon merolae, G. sulphuraria*, and *P. yezoensis*), chlorophytes (*C. reinhardtii, V. carteri, C. vulgaris, D. salina, C. subtellipsoidea, O. lucimarinus*, and *M. pusilla*), and streptophytes (*K. nitens*), led to the identification of a single ESL protein in the genome of *K. nitens*. In the genomes of 50 embryophytes, a total of 526 sequences were identified, and mosses and lycophytes have <5 ESL copies (2 for *Marchantia polymorpha*, 3 for *Physcomitrella patens*, 1 for *Sphagnum fallax*, and 2 for *Selaginella moellendorffii*). Gymnosperms and angiosperms have 5 to 19 ESL genomes, with a few exceptions of those having fewer ESL copies (*S. polyrhiza*: 3, *P. dactylifera:* 4, and *T. pratense*: 4) or a very high number of copies (*B. rapa*: 30, *L. usitatissimum*: 30, and *E. grandis*: 37). These data suggest that ESL transporters could have appeared with streptophytes and have considerably diversified in Embryophyta (Figure 1 and Supplementary Table 1).

**Figure 2:**
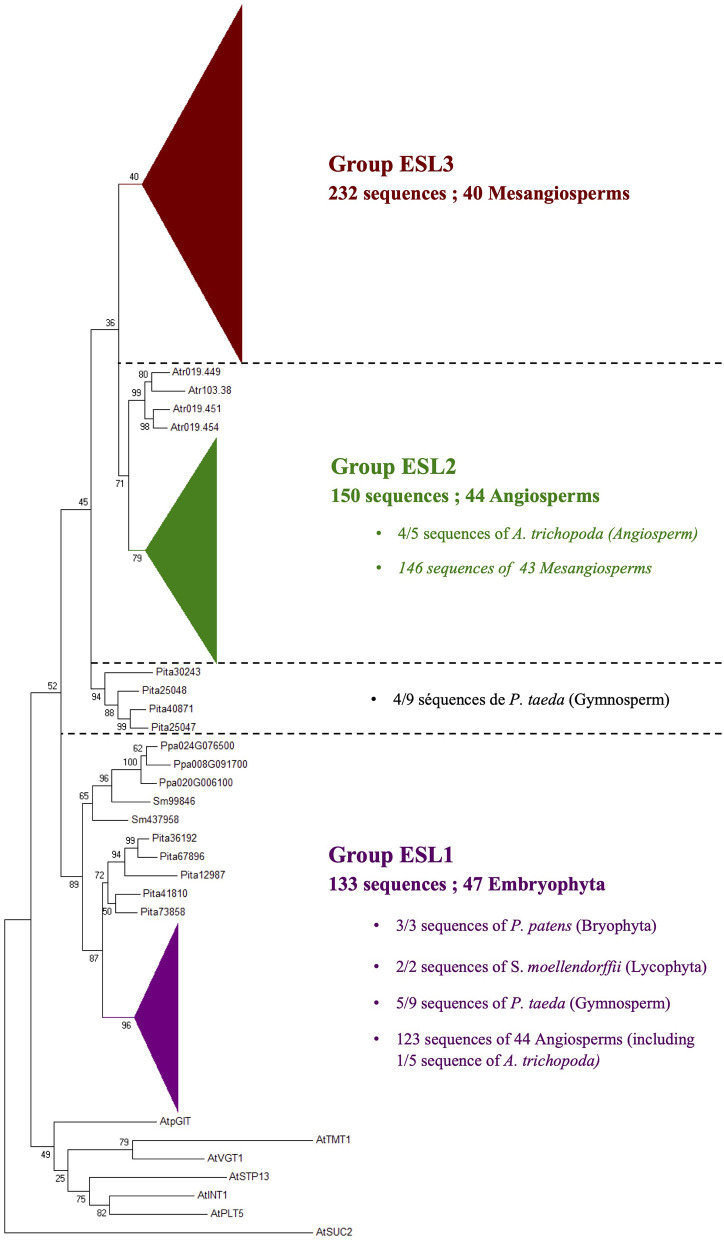
The maximum likelihood phylogeny of ESL sugar transporter proteins. The tree was produced by aligning 519 amino acid ESL sequences identified in 47 Embryophyta using ClustalW and was then built using the software Molecular Evolutionary Genetics Analysis (MEGAv6, Tamura et al., 2013). Jones–Taylor–Thornton (JTT) amino acid substitution model was used and the bootstrap consensus tree was inferred from 1,000 replicates. The percentage of replicate trees in which the associated taxa clustered together in the bootstrap test is shown next to the branches. The tree is drawn to scale, with branch lengths measured in the number of substitutions per site. *A. thaliana* sucrose carrier 2 (AtSUC2), *A. thaliana* polyol transporter 5 (AtPLT5), *A. thaliana* inositol transporter 1 (AtINT1), *A. thaliana* sugar transport protein 13 (AtSTP13), *A. thaliana* vacuolar glucose transporter 1 (AtVGT1), *A. thaliana* tonoplastic monosaccharides transporter 1 (AtTMT1), and *A. thaliana* plastidic glucose transporter 1 (AtpGlT). For the annotation of other sequences, see Supplementary Table 1.

**Figure 3:**
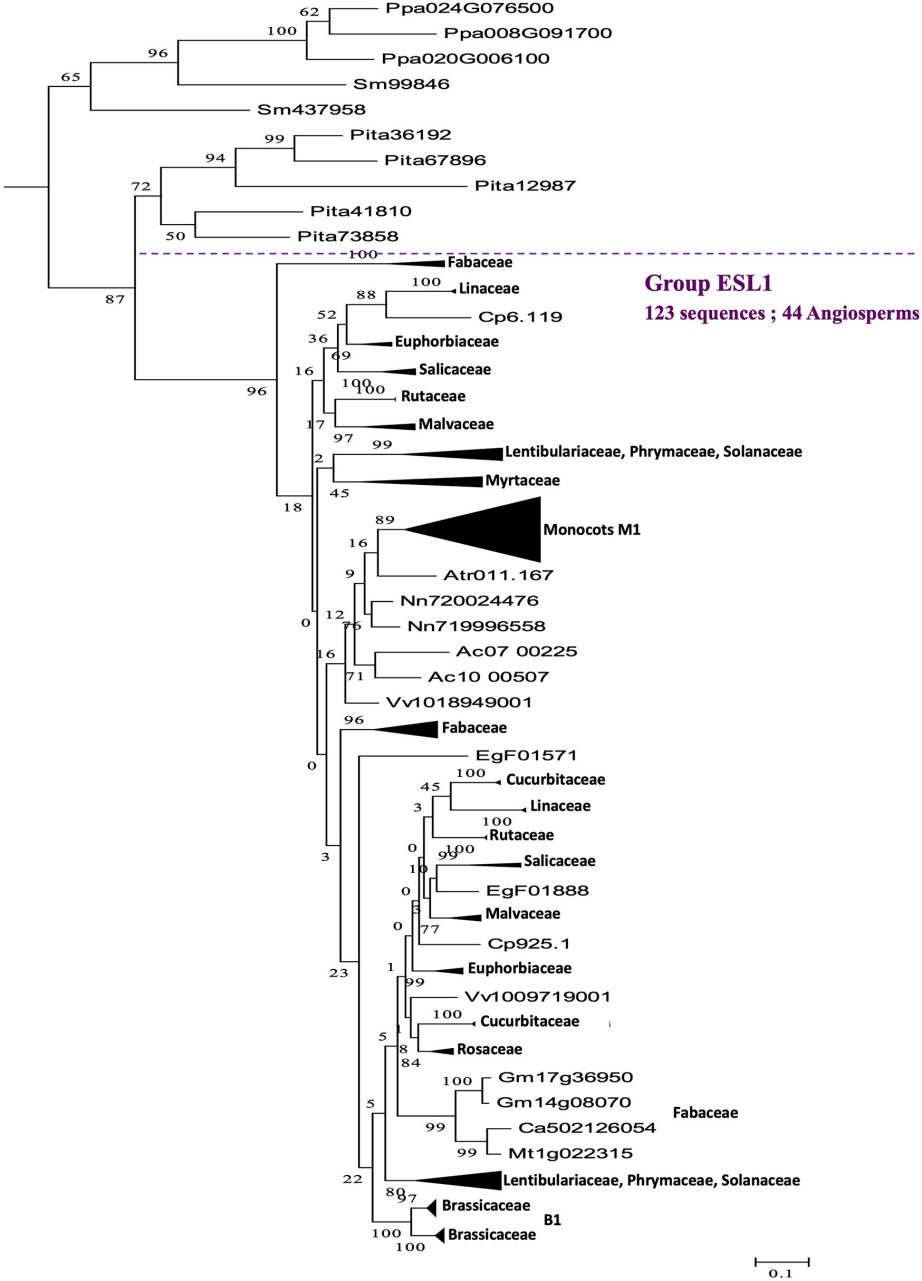
The maximum likelihood phylogeny of ESL sugar transporter proteins: The details of group ESL1. The tree was produced by aligning 519 amino acid ESL sequences identified in 47 Embryophyta using ClustalW and was then built using the software Molecular Evolutionary Genetics Analysis (MEGAv6, Tamura et al., 2013). JTT amino acid substitution model was used and the bootstrap consensus tree was inferred from 1,000 replicates. The percentage of replicate trees in which the associated taxa clustered together in the bootstrap test is shown next to the branches. The tree is drawn to scale, with branch lengths measured in the number of substitutions per site. For the annotation of sequences, see Supplementary Table 1.

A phylogenetic analysis performed on the 519 protein sequences isolated from 47 Embryophyta shows that the ESL proteins of angiosperms can be divided into three main groups, namely ESL1, ESL2, and ESL3. The group ESL1 (Figure 2) is supported by a high bootstrap value (96%) and contains 44 angiosperms with 123 sequences, including 1 of the 5 sequences of *A. trichopoda*. All the sequences identified in moss and fern (the tree sequences of *P. patens* ans the two sequences od *S. moellendorffii*) and 5 of the 9 sequences of the gymnosperm *P. taeda* are located at the basis of this group. Altogether, the 133 ESL protein sequences (25.6% of all sequences) form a cluster wherein the 47 analyzed species are represented by at least 1 protein sequence. The sequences of moss and fern form a monophyletic group and show a similarity higher than those of *P. taeda* and angiosperms. In the groupe ESL1 (Figure 3), monocots sequences form a monophyletic group supported by a bootstrap value of 89%. One sequence of *A. trichopoda*, two of *N. nucifera*, two of *A. caerulea* and one of *V. vinifera* are located at the basis of this monocots group. This observation suggests that monocots ESL1 are related to ESL of basal angiosp, basal eudicots and vitales. On the contrary, asterid sequences are split into two distinct groups, including both Solanaceae, Phrymaceae, and Lentibulariaceae, whereas for eurosid sequences, at least, two separated groups for most botanical families can be observed and most are supported by high bootstrap values. Fabaceae sequences are split into three groups, and a single group is observed for Rosaceae and Myrtaceae. According to Figure 2, the four other sequences of the gymnosperm *P. taeda* are located at the basis of groups ESL2 and ESL3, and no sequences of moss and fern are present. This suggests a first diversification event in the common ancestor of seed plants.

**Figure 4:**
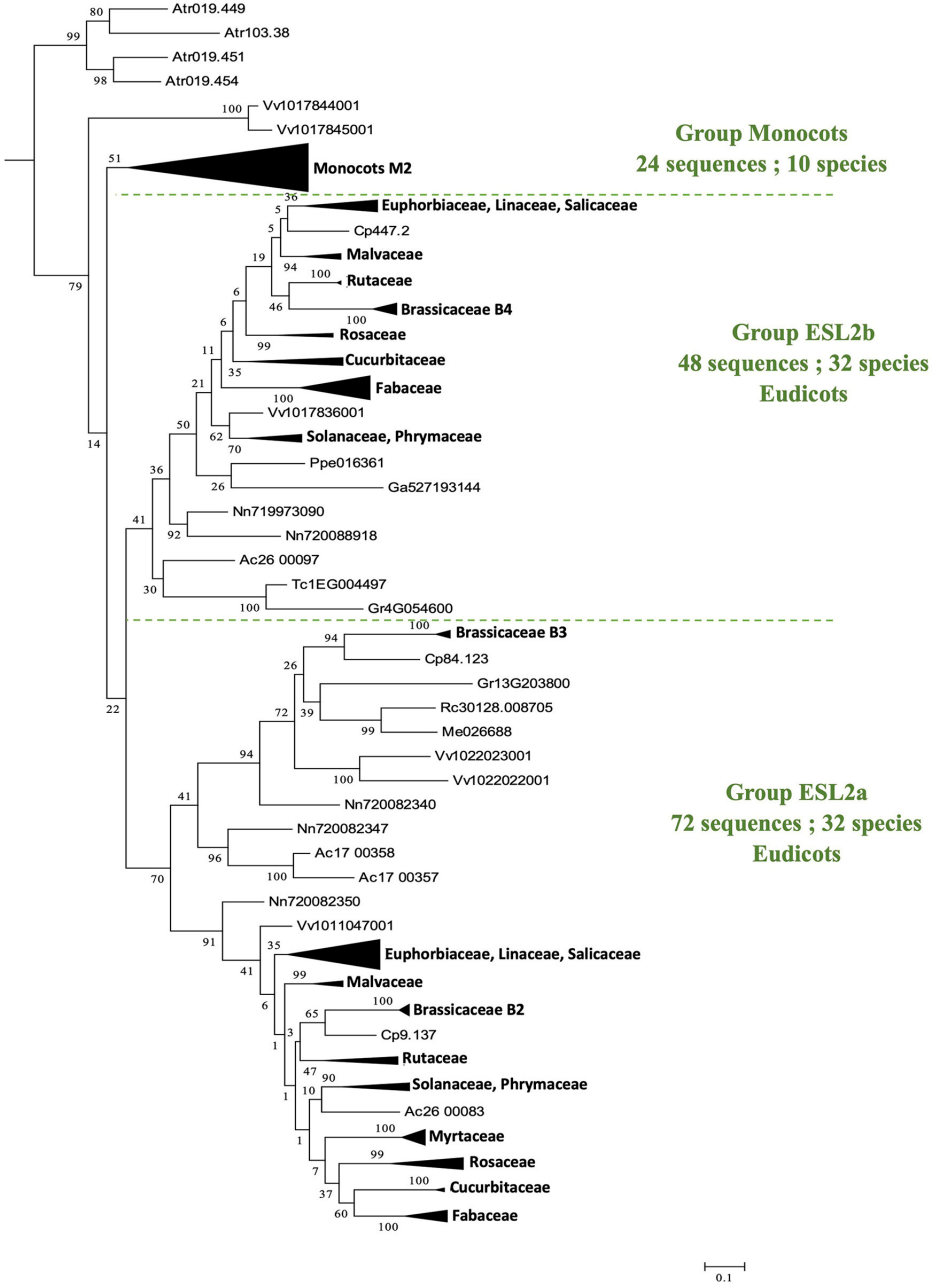
The maximum likelihood phylogeny of ESL sugar transporter proteins: The details of group ESL2. The tree was produced by aligning 519 amino acid ESL sequences identified in 47 Embryophyta using ClustalW and was then built using the software Molecular Evolutionary Genetics Analysis (MEGAv6, Tamura et al., 2013). JTT amino acid substitution model was used and the bootstrap consensus tree was inferred from 1,000 replicates. The percentage of replicate trees in which the associated taxa clustered together in the bootstrap test is shown next to the branches. The tree is drawn to scale, with branch lengths measured in the number of substitutions per site. For the annotation of sequences, see Supplementary Table 1.

The group ESL2 (Figure 2) is formed by 150 sequences of angiosperms (28.9% of all sequences). It is noteworthy that the 4 sequences of *A. trichopoda* are located at the basis of a mesangiosperm group (a bootstrap value of 79%), which contains the 146 sequences identified in the genomes of 43 species. In this group (Figure 4), monocots and eudicots separate clearly into two specific groups. The monocot group (monocots G2) contains 24 sequences (10 species). The eudicot group can be subdivided into two main subgroups: ESL2a (72 sequences, 32 species, excluding *L. japonicus*) and ESL2b (48 sequences, 32 species, *excluding E. grandis*). In each group, the protein sequences separate mainly according to the botanical families. There are two groups of ESL for Fabaceae, Rosaceae, Cucurbitaceae, Rutaceae, Malvaceae, Malpighiales, and Asterids, and three groups of ESL for Brassicaceae.

**Figure 5:**
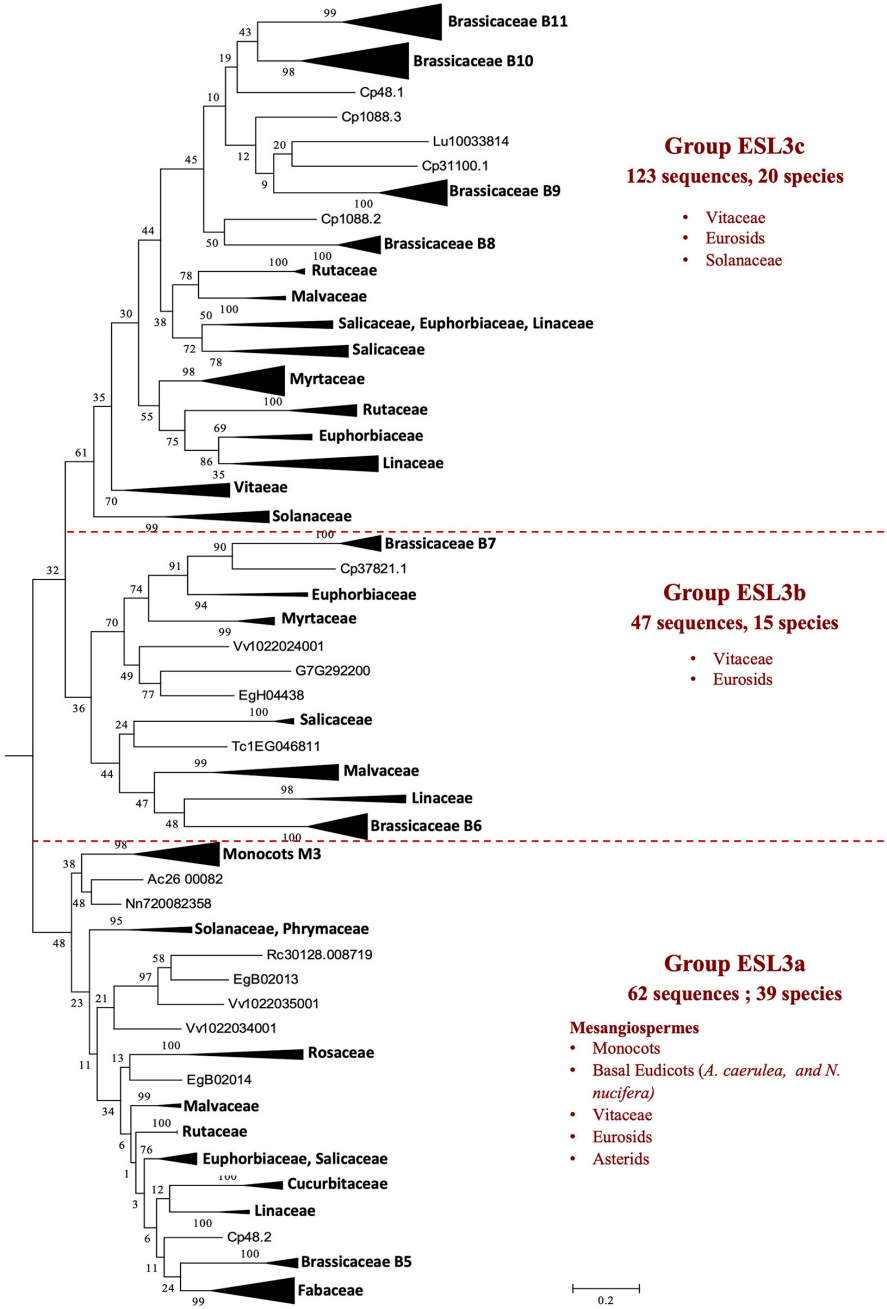
The maximum likelihood phylogeny of ESL sugar transporter proteins: the details of group ESL3. The tree was produced by aligning 519 amino acid ESL sequences identified in 47 Embryophyta using ClustalW and was then built using the software Molecular Evolutionary Genetics Analysis (MEGAv6, Tamura et al., 2013). JTT amino acid substitution model was used, and the bootstrap consensus tree was inferred from 1,000 replicates. The percentage of replicate trees, in which the associated taxa clustered together in the bootstrap test is shown next to the branches. The tree is drawn to scale, with branch lengths measured in the number of substitutions per site. For the annotation of sequences, see Supplementary Table 1.

The group ESL3 (Figure 2) is specific to mesangiosperms as no sequences of *A. trichopoda* (one of the earliest divergent lineages within angiosperms) are present. This group contains the 232 sequences of 40 species (44.7% of all sequences). *S. polyrhiza* (one of the earliest divergent lineages within monocots), *O. sativa*, and *G. aurea* are not represented in this group. The group ESL3 can be subdivided into three subgroups, namely ESL3a, ESL3b, and ESL3c (Figure 5). The subgroup ESL3a contains 62 sequences (11.95% of all sequences) from 39 mesangiosperms. One monocot-specific group (monocots G3) is related to a sequence of the two basal eudicots: *A. caerulea* and *N. nucifera*. All Pentapetalae (Vitaceae, Eurosids, and Asterids) form a single group in which the sequences are separated according to the botanical families. The subgroup ESL3b contains the 47 sequences (9.05% of all sequences) identified in *V. vinifera* and 14 Eurosids, and the subgroup ESL3c contains 123 sequences (23.7% of all sequences) from *V. vinifera*, Solanaceae (*S. lycopersicum* and *S. tuberosum*), and 17 Eurosids.

In conclusion, the distribution of ESL sequences in the three main ESL groups varies according to the botanical groups, and four distribution types can be observed (Supplementary Table 3): (1) The group ESL1 contains most of the sequences of Poaceae (49%) and Lamiales (58%) and is closely related to half of the sequences of *P. taeda* (56%) and to all the sequences of *P. patens* and *S. moellendorffii*. (2) The group ESL2 contains the highest number of ESL sequences of *A. trichopoda* (Amborellaceae, 80%), *A. corulea* (Ranunculaceae, 57%), *N. nucifera* (Nelumbonaceae, 63%), most of the Fabaceae (39%), all Rosaceae (53%), and Cucurbitaceae (43%). (3) For *V. vinifera* (Vitaceae 56%), all Malpighiales (52%), *E. grandis* (Myrtaceae, 59%), Rutaceae (52%), Brassicales (71%), and Malvaceae (48%), the highest number of proteins is found in the group ESL3. This high number is generally correlated with the presence of a high number of sequences in group ESL3c (55 to 81% of ESL3 sequences) except for malvaceae and euphorbiaceae. (4) For Solanales, the groups ESL1 and ESL3 contain the same number of sequences (38% each), which is more than the group ESL2 (24%). In monocots and Fabaceae, this distribution varies according to the species.

### Tandem Duplications Are at the Origin of the Expansion of the ESL Family in Some Monocots and Eudicots

**Figure 6:**
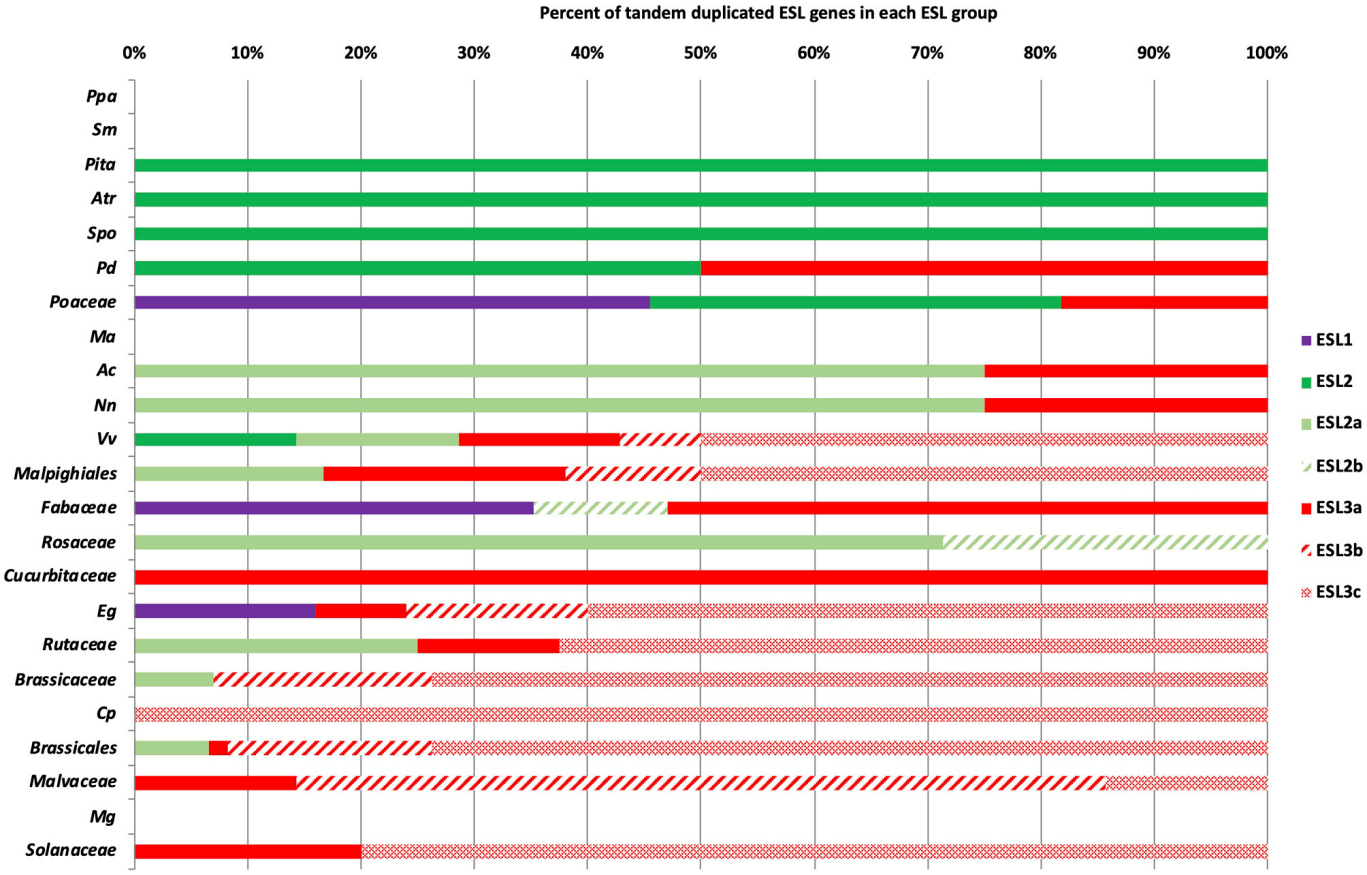
The distribution of tandem duplicated *ESL* genes in the different ESL groups. % represents the percentage of tandem duplicated genes relative to the total number of *ESL* per species or botanical groups. Purple is for *ESL1*, green for *ESL2*, and red for *ESL*3. For abbreviations see Supplementary Data 1.

**Figure 7:**
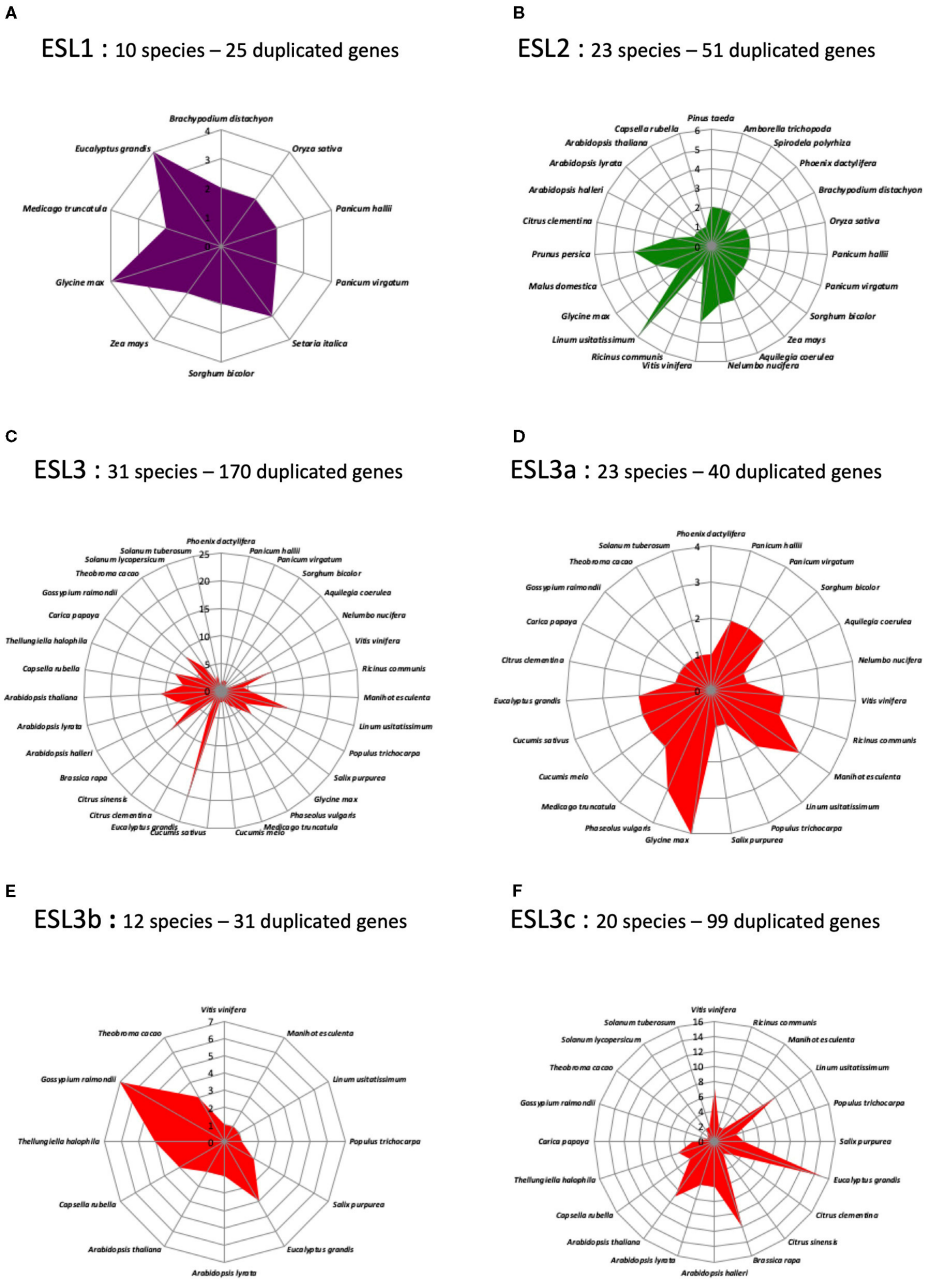
The distribution of the tandem duplicated *ESL* genes in the different ESL groups. Values correspond to the number of tandem duplicated genes for each species. **(A)** *ESL1*, **(B)** *ESL2*, **(C)** *ESL3*, **(D)** *ESL3a*, **(E)** *ESL3b*, and **(F)** *ESL3c*.

The genome analysis has revealed the presence of tandem duplicated genes for many species, which could explain the heterogeneous distribution of ESL into three ESL groups. To test this hypothesis, we determined the number of tandem duplicated *ESL* genes for each species and studied their distribution. *L. japonicus* and *G. aurea* were not included in this analysis (Figure 6 and Supplementary Table 3). For a few species, including the moss *P. patens*, the fern *S. moellendorffii*, the monocot *M. acuminate*, and the asterid *M. guttatus*, no tandem duplicated gene was identified. This could be in agreement, at least for the moss and the fern, with a few numbers of *ESL* genes found in their genomes. The gymnosperm *P. taeda*, the basal angiosperm *A. trichopoda*, and the basal monocot *S. polyrhiza* (alismatales) only have two duplicated *ESL2* genes. For monocots, *P. dactylifera* (arecales) contains two duplicated genes (one *ESL2* and one *ESL3a*), whereas in Poaceae, all species have three to six tandem duplicated *ESL* belonging to *ESL1* and/or *ESL2*. In addition, *P. hallii, P. panicum*, and *S. bicolor* have two duplicated *ESL3*. Therefore, Poaceae mainly have *ESL1* (45%) and *ESL2* (36%) duplicated genes. For eudicots, only three species have duplicated ESL1: *E. grandis* (Myrtales) with four duplicated *ESL1* (16% of the *EgESL*) and two Fabaceae, *G. max* and *M. truncatula*, which have two and four tandem duplicated *ESL1*, respectively, representing 35% of the total Fabaceae sequences. The basal eudicots, *A. coerulea* and *N. nucifera* have more than 70% of the duplicated *ESL2* genes and two Rosaceae only *ESL2* tandem genes. *V. vinifera, E. grandis*, Rutaceae, and Brassicaceae have more than 85% duplicated *ESL3*, whereas Cucurbitaceae, *C. papaya*, and Solanaceae only have duplicated *ESL3*. In sum, among the 509 analyzed *ESL*, 246 are tandem duplicated genes, including 25 *ESL1* from 10 species (Poaceae: 60% and eudicots: 40%), 51 *ESL2* from 23 species (eudicots: 63% and monocots: 29%), and 170 *ESL3* mostly from eudicots (96%). Furthermore, duplicated *ESL3* genes are not homogeneously distributed among the ESL3 subgroups. The subgroup ESL3a contains 33 duplicated eudicot genes (82.5%) and only 7 monocot genes (17.5%) that are identified in 23 species. The subgroups ESL3b and ESL3c have 31 and 99 eudicot duplicated genes, respectively, all from Pentapetalae species, including *V. vinifera*, Malpighiales, *E. grandis*, Brassicales, and Malvales. ESL3c also contains duplicated genes from Rutaceae and Solanaceae (Figure 7 and Supplementary Table 3). The group ESL3, which has the highest number of sequences, also contains the most tandem duplicated genes. In fact, we observed a good correlation between the number of tandem-duplicated genes and the number of *ESL* genes per species (Supplementary Figure 1A), which is not surprising. A similar correlation is observed between the number of duplicated *ESL3* and the number of *ESL* genes per species as well as the number of eudicot *ESL* (Supplementary Figures 1B,C), which makes sense as eudicot species are more represented in this study. However, a correlation is also found between the number of duplicated *ESL3c* and the number of *ESL3* genes per species (Supplementary Figures 1D,E), indicating that a high number of *ESL3c* (123 genes) is presumed due to numerous eudicot tandem gene duplications, which have occurred in 20 species.

### *ESL* Genes From Monocots and Brassicaceae Present Specific Gene Structures

**Figure 8:**
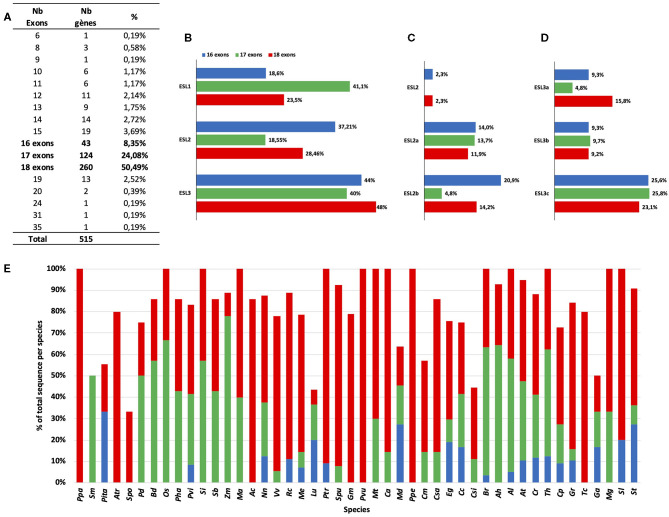
The percentage and the distribution of the different *ESL* gene structures (exons–introns). **(A)** The gene number per structure and the percentage of total *ESL*. **(B)** The distribution of the three main structures in the three ESL groups (% is related to the number of ESL having the same type of structure and belonging to the same *ESL* group). **(C)** Distribution amount of the *ESL2* group. **(D)** Distribution amount of the *ESL3* group. **(E)** The percentage of each type of structure per species.

To identify the potential structural specificities for *ESL* genes, we performed a comparative analysis of the intron/exon arrangement of 515 sequences from 46 species (*L. japonicus* was excluded from this analysis as no CDSs could be recovered on the *Lotus* genome website). As shown in Figure 8A, the number of exons present in the *ESL* genes varies from 6 to 35. However, 14 exon/intron arrangements with each representing <4% of the total number of sequences might arise from incomplete sequences or erroneous CDS or represent the exceptional structures that would only concern a small number of ESL sequences. By contrast, the arrangements with 16, 17, and 18 exons have been identified in 8.35%, 24.08%, and 50.49% of the *ESL* sequences, respectively.

**Figure 9:**
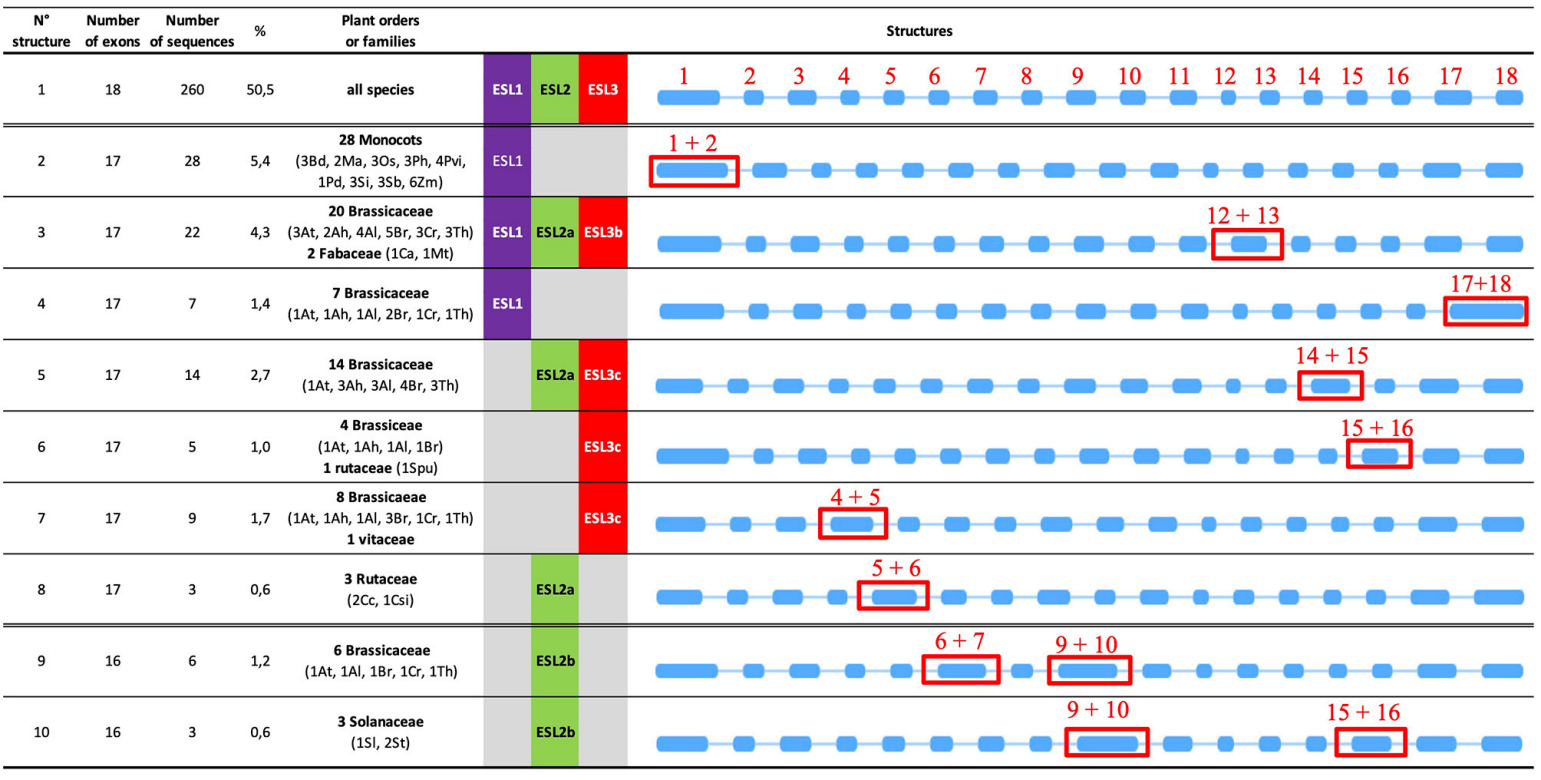
Major types of introns/exons structure identified in *ESL* genes. The blue rectangles represent the exons and the lines the introns. The introns are represented with a scale that does not take into account their real length. Red numbers indicate the exons and the red rectangles highlight the differences. For each structure, the number of exons and the number of sequences are indicated as well as the percentage of total ESL sequences and the botanical groups in which they have been detected.

The structure with 18 exons, named as structure 1 (Figures 8, 9), is the most widespread structure and is present in 260 embryophyte sequences (50.49%). It is found in all species except the fern *S. moellendorffii*. It is the only one found in the sequences of *P. patens* (moss), *P. vulgaris* (Fabaceae), and *P. persica* (Rosaceae). It is present in more than 80% of the sequences of the basal angiosperm *A. trichopoda*, the basal eudicot *A. coerulea*, the salicaceae *P. trichopoda* and *S. purpurea*, the Fabaceae *C. arietinum*, the Malvaceae *T. cacao*, and the Solanaceae *S. lycopersicum*. For the other species, it represents 20–80% of the *ESL* sequences, except in the cases of monocot *Z. maize*, the linaceae *L. usitatissimun*, and the lentibulatiaceae *G. aurea*, which have this structure for <20% of the sequences (Figure 8E). About 48% of the *ESL* sequences with this 18-exon structure are *ESL3* genes, including *ESL3c* being 23% (Figures 8B–D).

The arrangements with 17 exons are less widespread and have been identified in 124 embryophyte sequences (24.08%). They are present in more than 35% of the sequences of the fern *S. moellendorffii*, the Poaceae and the Brassicacea except for the cases of *P. virgatum* (33.3%), *and C. rubella* (29.4%). For *M. trucatula* and *M. guttatus*, they represent 30 and 33%, respectively, whereas, for all other species, they represent between 5.6 and 25% of the sequences. Genes with 17-exon arrangements are equally split into *ESL1* (41.1%) and *ESL3* (40%) groups, whereas only 18.55% are *ESL2* genes. In the *ESL3* group, a quarter of them are *ESL3c* (Figures 8A–D). In fact, 7 different structures with 17 exons (named as structures 2–8) were identified (Figure 9). Structure 2 should result from the merging of exons 1 and 2 relative to structure 1. It is specific to the monocot *ESL1* as it was identified in 28 monocot sequences from 9 Poaceae and from *P. dactilyfera* (arecaceae) and *M. acuminata* (musaceae). Only the *ESL* from the basal monocot *S. polyrhiza* does not have such a structure. Five 17-exon structures are more or less Brassicaceae specific. Structure 3 (the merging of exons 12 and 13) was identified in 22 sequences essentially from Brassicaceae (20 sequences) and Fabaceae (2 sequences) and concerned *ESL1, ESL2a*, and *ESL3b* genes. Structure 4 (the merge of exons 17 and 18) is specific to Brassicaceae *ESL1* as it is found in only seven *ESL1*. Structure 5 (the merge of exons 14 and 15) is specific to Brassicaceae and concerns 14 *ESL2a* and *ESL3c* genes. Structure 6 (the merging of exons 15 and 16) is specific to *ESL3c* and was identified in four Brassicaceae and in one *S. purpurea* (Rutaceae) genes. Structure 7 (the merging of exons 4 and 5) is specific to *ESL3c* and is identified in eight Brassicaceae and in one *V. vinifera* genes. Finally, the structure 8 (the merging of exons 5 and 6) is specific to *ESL2a* and is identified in three Rutaceae genes.

The arrangements with 16 exons are less abundant. They have been identified in 43 sequences (8.35%) from 20 species (Figures 8A–D). They are more present in *ESL2* (37.21%) and in *ESL3* (44%), especially in *ESL2b* (20.9%) and *ESL3c* (25.6%). They represent 33% of the *ESL* of *P. taeda*, 27% of those of *M. domestica* and *S. lycopersicum*, 20% of *S. tuberosum* and *L. usitatissimum*, and 3–12.5% of the *ESL* of Brassicaceae. Contrary to 17- and 18-exon structures, only the two types of 16-exon structures could be identified (Figure 9). Structure 9 (the merging of exons 6 and 7 as well as 9 and 10) is specific to *ESL2b* Brassicaceae and concerns six sequences in Brassicaceae. Structure 10 (the merging of exons 9 and 10 as well as 15 and 16) is observed for the three sequences of *ESL2b* of Solanaceae.

A striking result is that the *ESL1* from monocots have a 17-exon structure (structure 2), whereas *ESL2* and *ESL3* have mainly an 18-exon structure (structure 1). Furthermore, 17-exon structures are highly represented in Brassicaceae, of which five structures have been identified, including one specific to *ESL1* (structure 4), one specific to *ESL2a* (structure 8), two specific to the *ESL3c* (structures 6 and 7), and two others with less specificity (structures 5 and 3), to which a 16-exon structure specific to *ESL2b* is added (structure 9). Thus, we sought to determine whether there is a correlation between the observed structures and the different subgroups of monocots and Brassicaceae identified in the phylogenetic analysis, to highlight a possible link between *ESL* evolution and functional differences. In the phylogenetic analysis, 3 monocots (named as Mx) and 11 Brassicaceae (named as Bx) monophyletic groups were identified (Figures 2–5 and Supplementary Figure 2). Monocot 17-exon structure is only found in the group M1. Meanwhile, 18-exon structures are the majority in M2 and M3 groups. For the *ESL1* of Brassicaceae, the group B1 contains 15 sequences and can be divided into two subgroups each of which containing at least one sequence of the six species and characterized either by the 17-exon structure 3 or 4. Brassicaceae *ESL2a* genes are divided into two subgroups B2 and B3 having six *ESL* with 17-exon structure 5 and four *ESL* with the 17-exon structure 3, respectively. For *ESL2b* genes, the group B4 contains six sequences having the 16-exon structure 9. For *ESL3a*, the group B5 contains five sequences with the 18-exon structure 1. *ESL3b* genes are divided into two subgroups B6, containing 13 sequences with the 18-exon structure 1, and B7, containing eight sequences with the 17-exon structure 3. *ESL3c* genes are split into four subgroups: B8 containing nine sequences with the 18-exon structure 1, B9 may be subdivided into two parts: one characterized by the 17-exon structure 7 and the other containing two different structures, the 17-exon structure 7 and the 18-exon structure 1, B10 is formed by 13 sequences with the 17-exon structure 5 and B11 contains 18 sequences characterized by the 18-exon structure 1. In summary, the *ESL* genes that belong to the same Brassicaceae group present the same gene structure except the cases of groups B1 and B9 for which, two or three structures are present, certainly because they could be divided into subgroups.

### ESL Proteins From Monocots and Brassicaceae Present Conserved Protein Motifs

**Figure 10:**
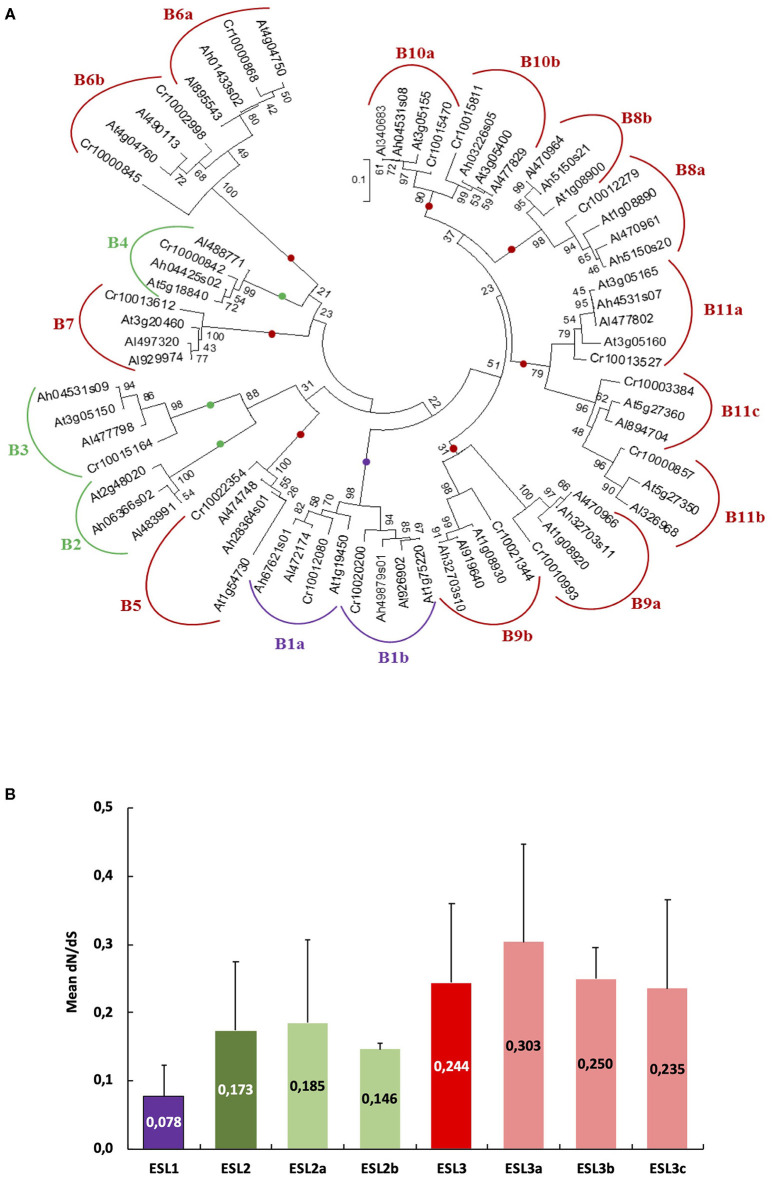
Nucleotide substitution analysis *(dN/dS)* for *Arabidopsis ESL*. **(A)** Phylogenetic tree produced *via* the alignment, using ClustalW, of 69 nucleotide ESL sequences from *Arabidopsis halleri, Arabidopsis lyrata, Arabidopsis thaliana*, and *Capsella rubella* used as an outgroup. The tree was built by using the software Molecular Evolutionary Genetics Analysis (MEGAv7, Tamura et al., 2013). JTT amino acid substitution model was used and the bootstrap consensus tree was inferred from 1,000 replicates. Purple is for *ESL1*, green for *ESL2*, and red for *ESL3*. **(B)** The means of the dN/dS values in each ESL group. Group ESL1 = 8 sequences, group ESL2 = 10 sequences, group ESL2a = 7 sequences, group ESL2b = 3 sequences, group ESL3 = 48 sequences, group ESL3a = 4 sequences, group ESL3b = 11 sequences, and group ESL3c =33.

To identify the common features and differences between ESL proteins and other sugar transporters, we used the MEME website search program and compared the protein domains in 193 ESL sequences (67 monocots and 126 Brassicales), 197 MSTs, and 31 SUCs. We have identified (Supplementary Table 5) 2 motifs common to the 3 sugar transporter families, named as ST-1 and ST-2, found in 95 and 79% of the sequences, respectively, 2 motifs common to the STP, 10 motifs common to the tonoplast MSTs (TMT), and 6 motifs for polyol/MSTs (PLT). Furthermore, the eight protein motifs identified in MSTs were also present in 91–97% of the ESL sequences. All these motifs were already described in *Saccharum* (Zhang et al., 2021). 13 motifs (ESL-motif 1 - 13) might be specific to ESL as they were not identified in other MST and SUC carriers. ESL-motif 1 and ESL-motif 2 are present in 92% and 96% of the ESL sequences, which means the same in all ESL groups. All the monocot ESL1 have the ESL-motif 3 (RDSSVSALLCTLIVAL) and some of them, the ESL-motif 5 (MSFRDZESGGEDGGRT), and ESL-motif 6 (TSNRGGGGAGEESGSDHDGGLR). Among them, the ESL-motif 3 is the only one to be present in the Brassicales ESL1 proteins (Supplementary Figures 4, 5). The Brassicales ESL2 contain the ESL-motif 8 (VTEPLLQKERKEEDSE) and the ESL2b have in addition the ESL-motif 12 (REIKDVERGEIVNKVE), which was not detected in the ESL2 of monocots. Brassicales ESL3b are split into two groups B6 and B7 (Figure 10), which differ in the presence of ESL-motif 9 (SSSPSSSSSLLSEISNASTRPFVLAFTVGSC) and the absence of ESL-motif 7 (AMVJLSTSVAVC) in the group B6, whereas, in the group B7, ESL-motif 7, ESL-motif 8, and ESL-motif 13 (SDKGIRVNDGDGDGPV) are present. About 18% of the ESL3c are characterized by ESL-motif 11 (MEEQRSMEKGLLLKKN). Finally, ESL-motif 10 (RERKFPNEDAFLETGLSRKSPR) is specific to the group B9b (Figure 10) of ESL3c Brassicales.

### *ESL* Transporters Genes in *Arabidopsis* Genus Are Under Negative Selection Pressure

For Brassicaceae, we identified 11 groups of ESL proteins (B1–B11), the presence of 68 tandem duplicated genes, and 7 different intron/exon structures. To better understand the evolutionary mechanisms involved in the genesis of the *ESL* Brassicaceae family, we aimed at determining the selection regime experienced by the family. We performed an analysis of nucleotide substitutions or dN/dS ratio for the *Arabidopsis* genus, the only one for which in our study the *ESL* genes have been identified in three different species (*A. thaliana, A. halleri*, and *A. lyrata*). The *ESL* sequences identified in *C. rubella* (another Brassicaceae) were used as an outgroup. To determine the number of alignments to be performed before calculating dN/dS ratios, a tree was built with the 65 CDSs of the three *Arabidopsis* and *C. rubella* (Figure 10A). 18 groups, named as B1 to B11c, could be defined. They correspond to the 11 groups of Brassicaceae identified in the phylogenetic tree of protein sequences (Figures 2–5). Considering all sequences, the average dN/dS ratio is 0.205, with the values always lower than 1 and ranging from 0.021 to 0.677. This indicates that the rate of non-synonymous substitutions is lower than that of synonymous substitutions. These results suggest that the *ESL* sequences are under a strong purifying (negative) selection pressure, which tends to conserve the sequences and potentially their functions. However, small differences can be detected between the means of the dN/dS ratios relative to each *ESL* group (Figure 10B). The value of the dN/dS ratio for the group *ESL1* (0.078) is the lowest one, whereas the value of the group *ESL3* is the highest (0.244) and the values of the subgroups *ESL2a* (0.185) and *ESL2b* (0.146) are intermediate. Among the *ESL3* subgroups, *ESL3a* shows the highest value (0.303). This suggests that even if the *ESL* genes are under a purifying selection, the selection pressure might be less stringent for the *ESL3* genes than for another *ESL*, especially *ESL1*.

### Expression of Tandem Duplication Genes Presents Different Patterns in *Arabidopsis* Plant

**Figure 11:**
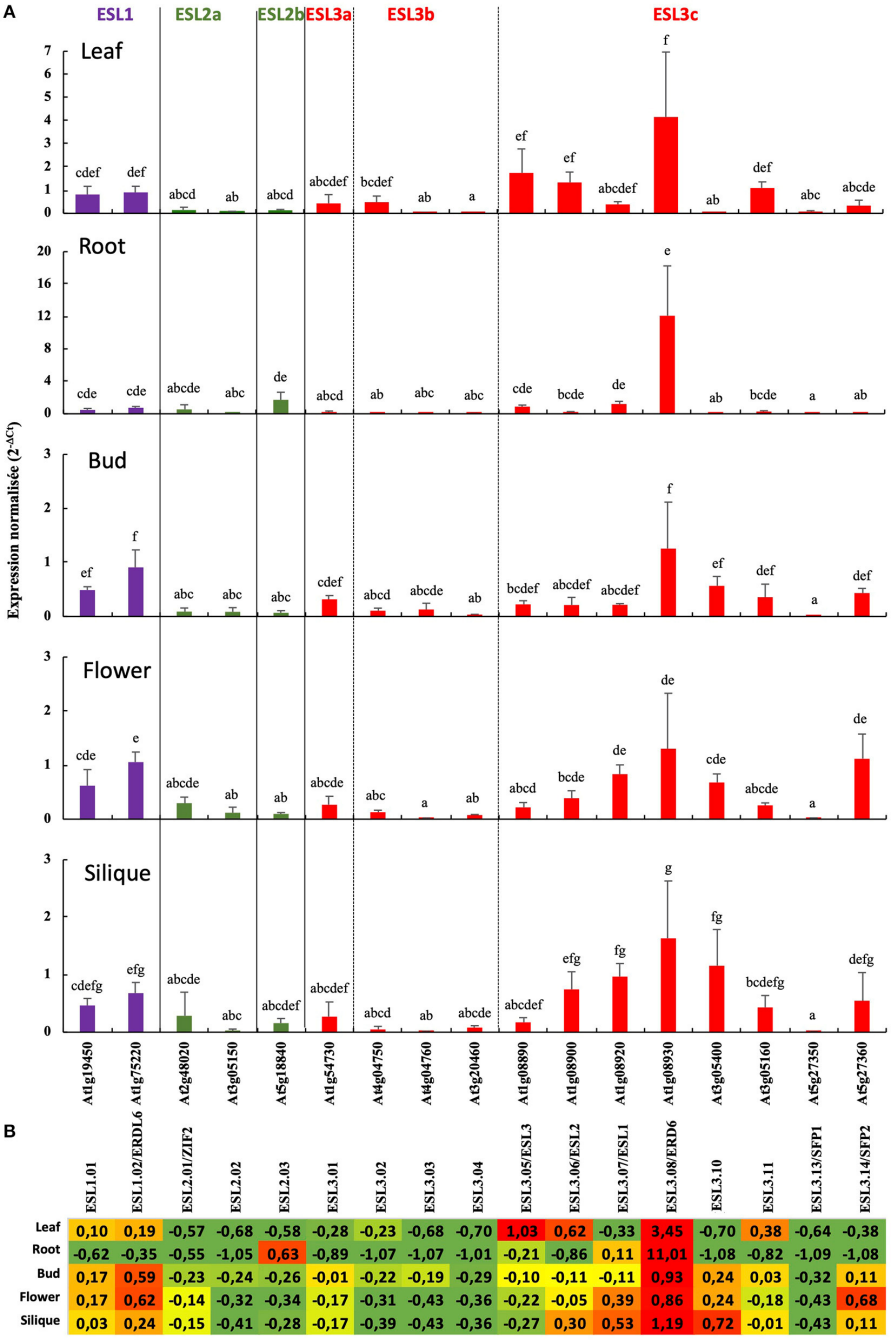
The relative expression of 17 *A. thaliana ESL* genes in different organs measured by real-time reverse transcription-PCR (qRT-PCR). **(A)** The 2^−ΔCt^ values are normalized according to *AtPP2a* expression and represent the mean of 3 biological repeats (ecartype). Statistical analysis was performed using the Kruskal-Wallis test and the multiple comparison test of Dunn corrected by Bonferroni. Values significantly different are indicated by distinct letters. Purple is for *ESL1*, green for *ESL2*, and red for *ESL3*. **(B)** The *ESL* gene expression relative to the average expression of all *ESL* in each tested organs. The values indicated for each gene correspond to the variation from the mean of 2^−ΔCt^ values determined for all *ESL* genes expressed in the considered organ. These means are equal to 0.71 in leaf, 1.09 in root, 0.32 in flower bud, 0.44 in flower, and 0.45 in silique. The relative expression values on a red, yellow or green background indicate that the expression of the considered gene is higher, equal or lower than the average of the mean expression of all *ESL*, in the indicated organ.

To determine whether some of the functions of *ESL* genes are conserved or, on the contrary, diverge, we analyzed the expression of the *ESL* genes from *A. thaliana*. First and foremost, we propose a new nomenclature for those genes according to the three main ESL groups we have identified in the present phylogenetic analysis. The nomenclature (*AtESL1.xx, AtESL2.xx*, and *AtESL3.xx*), as summarized in Table 1, emphasizes the *ESL* name of the group they belong to. As two tandem duplicated genes *AtESL3.09* (*At5g05155*) and *AtESL3.12* (*At5g05165*) could not be analyzed because no specific primers could be designed, the expression level of 17 genes was measured in leaves, roots, buds, flowers, and siliques, by using real-time reverse transcription-PCR (qRT-PCR) (Figure 11). The two *AtESL1* genes have median expression levels in the five tested organs, which range from 0.47 in roots and siliques to 0.82 in leaves for *AtESL1.01* and from 0.68 in siliques to 1.06 in buds for *AtESL1.02. AtESL1.02* is slightly more expressed than *AtESL1.01* in all organs. The three *AtESL2* genes present a lower expression level. *AtESL2.01/ZIF2* and *AtESL02.2* are constantly expressed in all organs, whereas *AtESL2.03* is more expressed in roots (1.72 ± 0.94) than in the other organs (0.11 ± 0.81). *AtESL3* genes present very diverse expression levels. *AtESL3.02, AtESL3.03, AtESL3.04*, and *AtESL3.13/SFP1* are the least expressed *ESL3* genes in all organs, and their expression level is equivalent to that of *AtESL2* genes. On the contrary, *AtESL3.08/ERD6* is the most expressed gene in all tested organs. Its expression level is 3.5 and 11 times more important in leaves and roots than the mean of the expression values for all *ESL* in the corresponding organs. *AtESL3.01* is expressed in all organs but shows a weaker expression in roots. The other *AtESL3c* present clear organ specificities: *AtESL3.05/ESL3* expression level is 3.5 times higher in leaves (1.74 ± 1.04) than in other organs (0.37 ± 0.34), *AtESL3.06/ESL2* is more expressed in leaves (1.33 ± 0.45) and siliques (0.74 ± 0.31), *AtESL3.07/ESL1* is more expressed in roots, flowers, and siliques than in leaves and buds, *AtESL3.10, AtESL3.11*, and *AtESL3.14/SFP2* are more expressed in siliques (1.16 ± 0.63), leaves (1.09 ± 0.26), buds (1.12 ± 0.46), respectively. The normalization of the total mean expression in each organ highlights that six *ESL* (*ESL3.08, ESL3.05, ESL3.06, ESL3.11, ESL1.02* and *ESL1.01*) are highly expressed in leaves, three (*ESL3.8, ESL2.03*, and *ESL3.07*) in roots, five (*ESL3.08, ESL1.02, ESL3.10, ESL1.01*, and *ESL3.14*) in buds, six (*ESL3.08, ESL3.14, ESL1.02, ESL3.07, ESL3.10* and *ESL1.01*) in flowers and five (*ESL3.08, ESL3.10, ESL3.07, ESL3.06*, and *ESL1.02*) in siliques.

**Table 1:** Nomenclature of *Arabidopsis thaliana* early response to dehydration six-like (*ESL*) genes.

**ESL group**	**Brassicaceae group**	**Gene ID**	**AtERD6-Llke x**	**New names**
ESL1	B1a	At1g19450	AtERD6-Like 4	ESL1.01
ESL1	B1b	At1g75220	AtERD6-Like 6	ESL1.02/ERDL6
ESL2a	B2	At2g48020	AtERD6-Like 7	ESL2.01/ZIF2
ESL2a	B3	At3g05150	AtERD6-Like 8	ESL2.02
ESL2b	B4	At5g18340	AtERD6-Like 16	ESL2.03
ESL3a	B5	At1g54730	AtERD6-Like 5	ESL3.01
ESL3b	B6a	At4g04750	AtERD6-Like 14	ESL3.02
ESL3b	B6b	At4g04760	AtERD6-Like 15	ESL3.03
ESL3b	B7	At3g20460	AtERD6-Like 13	ESL3.04
ESL3C	B8a	At1g08890	AtERD6-Like 1	ESL3.0S/ESL3
ESL3C	B8b	At1g08900	AtERD6-Like 2	ESL3.06/ESL2
ESL3C	B9a	At1g08920	AtERD6-Like 3	ESL3.07/ESL1
ESL3C	B9b	At1g08930	AtERD6	ESL3.08/ERD6
ESL3C	B10a	At3g05400	AtERD6-Like 12	ESL3.09
ESL3C	B10b	At3g05155	AtERD6-Like 9	ESL3.10
ESL3C	B11a	At3g05160	AtERD6-Like 10	ESL3.11
ESL3C	B11a	At3g05165	At3g05165	ESL3.12
ESL3C	B11b	At5g27350	AtERD6-Like 17	ESL3.13/SFP1
ESL3C	B11c	At5g27360	AtERD6-Like 18	ESL3.14/SFP2

**Figure 12:**
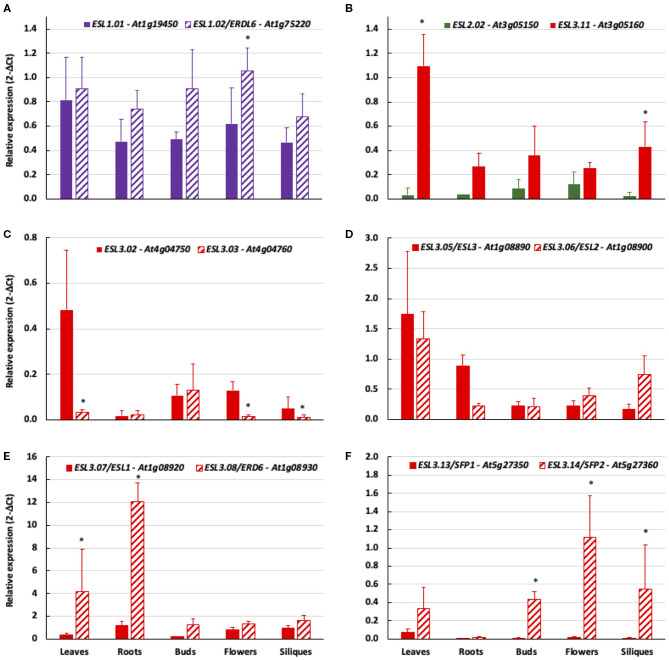
The comparison of the relative expression of tandem duplicated *ESL* genes in different organs measured by qRT-PCR. 2^−ΔCt^ values are normalized according to *AtPP2a* expression and represent the mean of three biological repeats (± ecartype). Statistical analysis was performed by using the Kruskal–Wallis test and the multiple comparison test of Dunn corrected by Bonferroni. Values significantly different are indicated by distinct letters. **(A)** *ESL1.01/ESL1.02*, **(B)** *ESL2.02/ESL3.11*, **(C)** *ESL3.02/ESL3.03*, **(D)** *ESL3.05/ESL3.06*, **(E)** *ESL3.07/ESL3.08*, and **(F)** *ESL3.13/ESL3.14*.

The bioinformatic analysis led to the identification of five tandem duplications (*ESL2.02/ESL3.11, ESL3.02/ESL3.03, ESL3.05/ESL3.06, ESL3.07/ESL3.08*, and *ESL3.13 /ESL3.14*). We therefore compared the expression patterns of tandem genes as well as that of the two *ESL1* (Figure 12). The expression level of *ESL1.01* and *ESL1.02* is similar in all organs even if *ESL1.01* is a little bit more expressed in flowers (Figure 12A). *ESL3.11* is higher expressed in most organs and especially in leaves (36x) and siliques (14x) than *ESL2.02* (Figure 12B). Compared to ESL3.03, *ESL3.02* is significantly more expressed in leaves (16x), flowers (10.7x), and siliques (5x) (Figure 12C) even if the expression level of both genes is weak. No significant difference was observed between *ESL3.05* and *ESL3.06*, which are expressed at the same level in all tested organs (Figure 12D). *ESL3.07* and *ESL3.08* have highly different expression levels in vegetative organs: compared to *ESL3.07, ESL3.08* is more highly expressed in leaves (10.6x) and roots (10x), whereas, in reproductive organs, this difference disappears as both are weakly expressed. On the opposite, *ESL3.13* and *ESL3.14* present more different expression levels in reproductive organs than in vegetative organs. Compared to *ESL3.13, ESL3.14* is 71.6x, 111x, and 91.6x times more expressed in buds, flowers, and siliques, respectively.

### *Cis*-Acting Elements Putatively Involved in the Transcriptional Regulation of *AtESL* Transporters

We performed a PLACE analysis on the 1-kb promoter region of each of the 19 *AtESL*, and found 186 *cis*-acting elements that were classified into three main categories: growth and development, hormone response, and stress response, the last ones are the most numerous ones (Supplementary Figure 3). 12 common motifs were found in all analyzed *ESL* genes (Supplementary Figure 3B). These motifs are highly repetitive (up to 27 copies), which might be due to their limited length (4–6 bases). Among the 12 *cis*-acting elements, some of the elements are able to regulate the expression in different plant organs such as leaves, pollen, and roots, and the rest of the elements are involved in hormonal signaling (cytokinin, gibberellic acid, and salicylic acid) or in response to biotic and abiotic environmental factors such as light and salinity. Interestingly, one motif (WBOXHVISO1: sequence TGACT) involved in sugar regulation, is present in all *ESL* promoters, in a few copies. *Cis*-elements regulating the expression in leaves or mesophyll cells are the most represented ones. In addition to the common *cis*-regulatory elements, we identified 30 *cis*-acting elements, which were only found in a single promoter, thereby suggesting expression specificity (Supplementary Table 4). Their length varied from 6 to 19 bp. Interestingly, among the 30 identified gene-specific motifs, 5 are only present in the *ESL3.08/ERD6* promoter and 4 in *ESL3.01* and *ESL3.05/ESL3*, whereas *ESL1.02/ERDL6, ESL2.02, ESL3.03, ESL3.04*, and *ESL3.06/ESL2* do not have specific elements. These motifs can be grouped into different functional categories, which are related to hormonal regulation (6), abiotic stresses (5), cellular development (6), nutrition (3), and plant growth and development (1).

**Figure 13:**
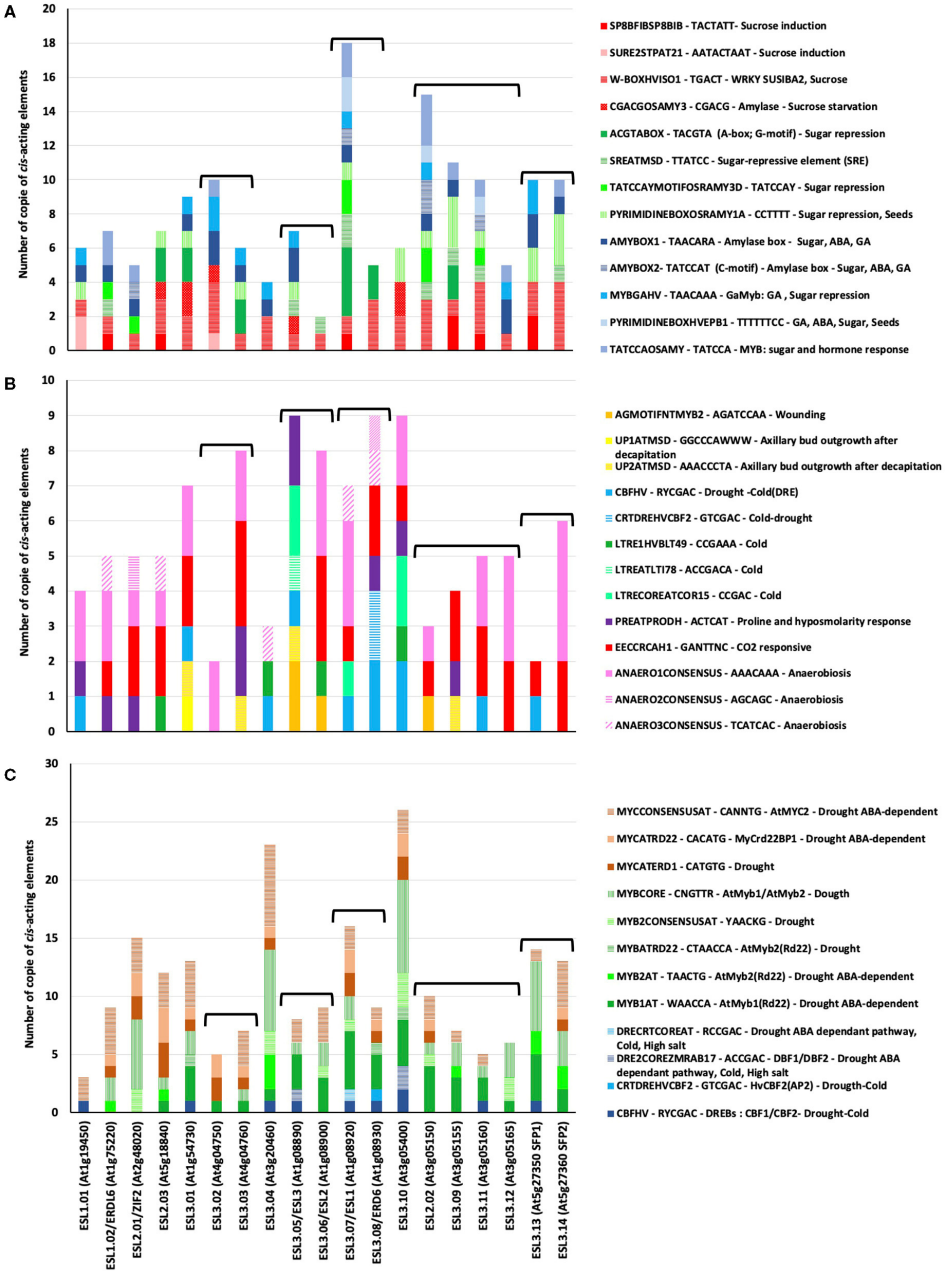
Sugar and stress *Cis*-regulatory elements found in the 1-kb promoter of *Arabidopsis thaliana ESL* genes. **(A)** *Cis*-elements involved in sugar regulation. **(B)** *Cis*-elements involved in abiotic stresses response. **(C)** *Cis*-elements involved in drought response.

*In silico* analysis of *AtESL* promoters resulted in the identification of the 13 motifs that are potentially involved in sugar-regulated transcription (Figure 13A). We found cis-acting elements involved in sucrose induction such as SP8 and WBOXHVISO1 (sequences enabling the binding of some WRKY-type proteins in the example of SPF and SUSIBA2), SURE boxes (SURE2STPAT21), and the CGACGOSAMY3. Four elements are involved in sugar repression (ACGTABOX, SREATMSD, TATCCAYMOTIFOSRAMY3D, and PYRIMIDINEBOXOSRAMY1A), and 5 *cis*-elements are involved in common hormonal and metabolic (sugar) signal perception such as the GARC complex comprising the AMYBOX1 and 2, the MYBGAHV for gibberellins (GAs) induction and sugar repression, the PYRIMIDINE boxes for GAs, ABA, and TATCCOSAMY, a sequence enabling the binding of MYB proteins. It is noteworthy that the number and the distribution of these sugar elements are really different between the tandem duplicated copies with the exception of *ESL3.13/SFP1* and *ESL3.14/SFP2* and the two *ESL1*.

We also found numerous *cis*-acting elements involved in diverse stress responses (Figure 13B). The two most abundant ones are the elements involved in anaerobiosis (ANAEROxCONCENSUS), which are found in all *AtESL* except *ESL3.05/ESL2, ESL3.09*, and *ESL3.13* and a CO_2_-responsive element (EECCRAH1) found in most *AtESL* with the exception of *ESL1.01, ESL3.02*, and *ESL3.05/ESL2*. Five elements involved in cold response are mainly present in the promoter of *ELS3* genes as well as three others involved in wounding and axillary bud outgrowth after decapitation. The number and the distribution of these elements are really different between *ESL1.01* and *ESL1.02* as well as the two tandem genes *ESL3.07/ESL1* and *ESL3.08/ERD6*. Differences could be observed between the two pairs of tandem genes: *ESL3.02* only has an ANAERO1CONSENSUS element, whereas *ESL3.03* also has *cis*-elements for hypo-osmolarity, CO_2_, and wounding responses. *ESL3.13/SPF1* has *cis*-elements involved in cold response, whereas *ES13.14/SFP2* has elements for CO_2_ response. Finally, we detected numerous drought-responsive *cis*-elements in all *AtESL* promoters (Figure 13C) such as DREB or element enabling either the binding of MYB or MYC transduction factors. The *cis*-elements that seem to be most abundant are the MYB motifs. Some differences can be observed between *ESL1.01* and *ESL1.02*, which does not have *cis*-acting elements for MYB but instead DREB elements, and the number of *cis*-acting elements in ESL3.07/ESL1 is higher than that found in ESL3.07/ERD6. This analysis suggests that *AtESL* might be differentially regulated by sugar, hormones, and diverse abiotic stresses such as drought and opens perspectives for future research.

## Discussion

The present study provides evidence for the early origin of ESL MSTs in streptophytes. This hypothesis is based on the fact that no ESL was identified in any of the chlorophyte (eight species) and rhodophyte (three species) genomes as well as in that of the brown algae *E. siliculosus*, whereas a single ESL was identified in the genome of *K. nitens* (Figure 1). It is noteworthy that a high stringency imposed in the search of ESL proteins could be restrictive for the identification of MSTs in algae displaying a low level of homology to Embryophyta ESL. However, our conclusion is supported by the fact that carriers similar to sucrose and other MSTs have been identified in algae. SUC homologs have been identified in the genomes of the characean algae *Chlorokybus atmosphyticus* (Reinders et al., 2012), rhodophytes (*C. merolae* and *G. sulphuraria*), and chlorophytes (*O. lucimarinus* and *O. tauri*) (Peng et al., 2014), even though some chlorophytes do not have SUC homologs, such as *C. reinhardtii* and *V. carterii* (Reinders et al., 2012). Close homologs of MSTs (HUP1, HUP2, and HUP3) have been found in the green algae *Chlorella* (Sauer and Tanner, 1989; Caspari et al., 1994; Stadler et al., 1995; Johnson et al., 2006). However, they are more closely related to the STP monosaccharide transporter subfamily than to the ESL. Taken together, these data suggest the existence of some hexoses and SUCs in algae and support the hypothesis that ESL carriers have arisen after sucrose and STP transporters.

The identification of an ESL transporter in *K. nitens* is in agreement with the generally accepted hypothesis that the ancestors of terrestrial plants are closely related to the charophytes (Lewis and McCourt, 2004; Leliaert et al., 2012; Timme et al., 2012). The analysis of the genomes of *K. flaccidum* (Hori et al., 2014) and *C. braunii* reveals that both organisms have acquired several plant genes, involved in the production of phytohormone, tolerance to a high-intensity light and to reactive oxygen species, as well as transcription factors (Hori et al., 2014; Nishiyama et al., 2018). This suggests that charophytes have already acquired the necessary features required for an adaptation to terrestrial life, such as the tolerance to drought and freezing, as demonstrated for some *Klebsormidium* species (Morison and Sheath, 1985; Elster et al., 2008; Nagao et al., 2008; Karsten and Holzinger, 2012). As characterized ESL carriers have been described to be regulated in response to environmental constraints (cold, drought, high salinity, temperature, wounding, and heavy metals), they may be involved in the adaptation of streptophytes to terrestrial environments.

**Figure 14:**
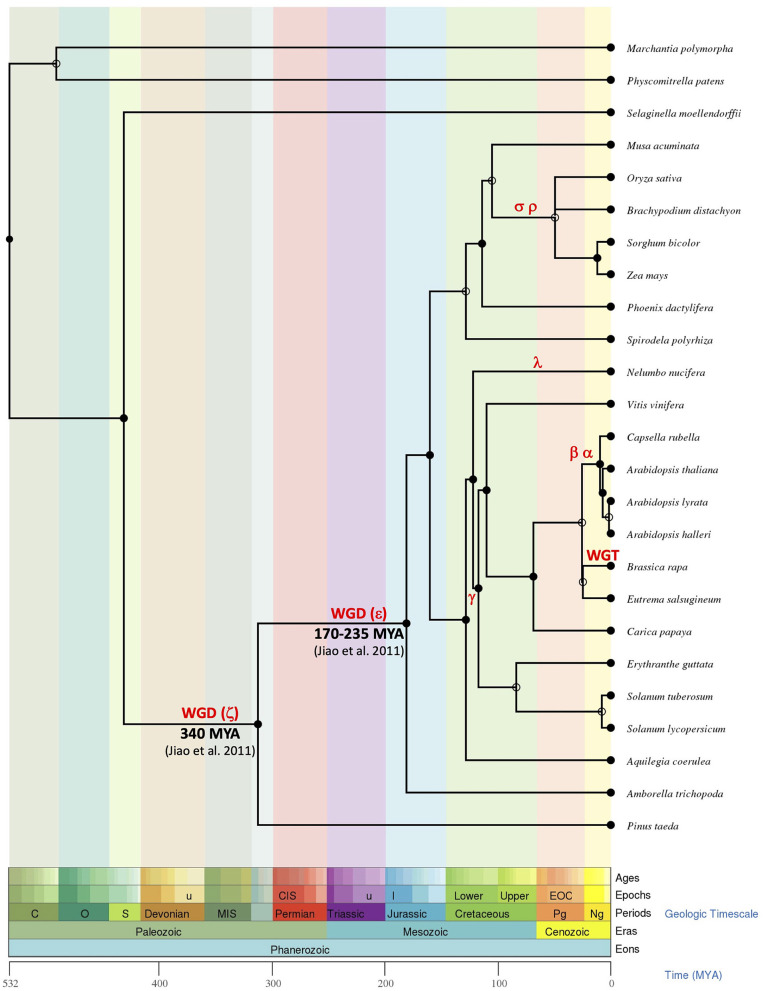
The phylogenetic tree inference of the evolution of *ESL* genes in the plant kingdom. All dates, periods, and evolutionary data used to rebuild the evolutionary history of the ESL family through the evolution of land plants were obtained from the Time Tree of Life (http://www.timetree.org, Hedges et al., 2006; Kumar et al., 2017). For readability reasons, only the most striking species have been considered. Major duplication events are indicated according to the literature.

The presence of ESL in all analyzed embryophytes genomes, with a lower number of copies in bryophytes and lycophytes (one for *S. fallax*, two for *M. polymorpha* and *S. moellendorffii*, and three for *P. patens*) than in seed plants (3–37 copies), suggests that ESL carriers have undergone a significant expansion and have become a large multigenic family in angiosperms, particularly in eudicots. Our phylogenetic analysis of 519 ESL proteins identified in the proteomes of 47 Embryophytes provides evidence for the existence of three ESL monophyletic groups, namely ESL1, ESL2, and ESL3 (Figure 2). Group ESL1 encompasses the sequences from each studied embryophyte, including all copies present in the genomes of *P. patens* (bryophyte), *S. moellendorffii* (lycophyte), and half of those of *P. taeda* (gymnosperm) (Figure 3). Thus, group ESL1 may represent the most ancestral ESL group. The fact that the sequences of gymnosperms and angiosperms are the only ones to split into two distinct groups and that the sequences of *P. taeda* are located on the basis of both ESL2 and ESL3 groups (Figures 2, 3) suggest a diversification in the common ancestor of seed plants (Figure 14). This event may match the most ancient whole genome duplication (WGD) named as paleopolyploidy (or ζ) which occurred approximately 340 MYA in the common ancestors of all spermaphytes (Jiao et al., 2011). Similarly, mesangiosperm sequences are split to form ESL2 and ESL3 groups, whereas the ESL sequences of the basal angiosperm *A. trichopoda* are present only on the basis of group ESL2 and are absent in group ESL3, which is therefore specific to mesangiosperms. These observations suggest a second diversification event in the common ancestor of the mesangiosperms (Figure 14), which could be related to the WGD ε described in the angiosperm lineage with the occurrence of 170–235 MYA (Jiao et al., 2011). The ESL3 group can be clearly divided into three subgroups, namely ESL3a, ESL3b, and ESL3c. The sequences of the monocots and of the basal eudicots: *A. coerulea* and *N. nucifera* only belong to the ESL3a and are excluded from the ESL3b and ESL3c groups, which thus represent Pentapetalae- (rosids and asterids) specific subgroups. This suggests a third diversification event in the common ancestor of the Pentapetalae (Figure 14), possibly corresponding to the whole genome triplication event (WGT) γ/1R that has been placed early in the evolution of eudicots evolution, around 117 MYA, before the separation of rosids and asterids and after the split of monocots and dicots (Jiao et al., 2012). This event witnessed the existence of an ancient hexaploidy for the eudicots. The absence of sequences of the basal eudicots in the groups ESL3b and ESL3c is in total agreement with the fact that the γ/1R triplication event has been placed after the divergence of the ranunculales and core eudicots (Jiao et al., 2012) and that the genome of *N. nucifera* has undergone another duplication event named λ (Ming et al., 2013). The fact that monocots are also not present in ESL3b and ESL3c groups is in agreement with the description of two important WGD, named ρ and σ, that have occurred before the divergence of Poaceae and are independent of the γ/1R event (Blanc and Wolfe, 2004; Paterson et al., 2004; Tang et al., 2010; D'Hont et al., 2012). Thus, we suggest that the ESL3b and ESL3c sequences, the only sequences from Vitaceae, Eurosids, and asterids, may be related to the γ*/*1R triplication event (Figure 14). However, ESL3b and ESL3c copies may also be the marks of more recent duplication events. Such events have been described for Salicaceae (Tuskan et al., 2006), Fabaceae (Cannon et al., 2005, 2006; Sato et al., 2008; Varshney et al., 2013) *M. domestica* (Velasco et al., 2010), and Malvaceae (Argout et al., 2011; Wang et al., 2012). For Brassicaceae, in addition to the γ/1R, 2 WGD, named as α/3R and β/2R (Bowers et al., 2003; Jiao et al., 2011), have been described to occur after the divergence of Brassicaceae and Caricaceae around 47 and 124 MYA, respectively (Kagale et al., 2014). The six Brassicaceae ESL groups observed in ESL3b and ESL3c groups might, therefore, be related to these three WGD events. In addition, the genus *Brassica* underwent a triplication (WGT) event around 7.9 and 14.6 MYA (Lysak et al., 2005; Beilstein et al., 2010), which could explain the high number of ESL genes (30 genes) identified in *B. rapa* genome. Segmental duplications may also explain the ESL diversification. It might be the case for *L. usitatissimum*, for which a specific WGD event detected around 5–9 MYA has been described, as well as numerous segmental duplications (Wang et al., 2012).

An interesting result is the observation of a high number of tandem duplications in the ESL subfamily. We identified 246 duplicated genes representing 47% of the 519 analyzed ESL. Their proportion varies with ESL groups. Furthermore, tandem duplicated genes are presented more in number in the *ESL3c* group compared with *ESL3a* and *ESL3b*. In this regard, we observed a good correlation between the number of tandem duplicated *ESL3c* genes and the total number of *ESL3* per species (Figure 7, Supplementary Figure 1, and Supplementary Table 3). *ESL3c* tandem duplications are dominant in *Vitis*, Eurosids, and Solanales but were not detected in Fabales, Rosaceae, Cucurbitales, and Phrymaceae. For Fabales, this is certainly correlated to a weaker expansion of *ESL* genes than those observed for other monosaccharide carrier subfamilies such as STP, PLT, and INT (Johnson et al., 2006; Doidy et al., 2019). *E. grandis* presents a remarkable expansion with 37 *ESL* genes among which 25 are genes in tandem repeats (67.6%). The observation is in agreement with the study of Myburg et al. (2014), who described in this species the largest number of genes in tandem repeats (34% of the total genes). These results suggest that tandem duplications are also involved in the expansion of the ESL family, in particular for *ESL3c* genes. The analysis of the phylogenetic distribution of tandem duplications in the seven Brassicales genomes (six Brassicaceae and one Caricaceae) suggests that those are specific to Brassicaceae and therefore are unrelated to genome doubling events alpha, beta, and to the specific Brassica WGT. Based on the Time Tree of Life, this translates into 25.6–68 MYA. It has been reported that tandem duplicated genes, which are retained in a lineage-specific manner, are mostly responsive to environmental biotic (Hanada et al., 2008) or abiotic (Zhang et al., 2016) stimuli. Likewise, the observation of a high number of tandem duplications for ESL suggests the involvement of these transporters in response to environmental cues, such as those already demonstrated for both abiotic (Kiyosue et al., 1998; Quirino et al., 2001; Yamada et al., 2010; Poschet et al., 2011; Remy et al., 2014) or biotic stresses (Breia et al., 2020) for few ESL.

The analysis of non-synonymous and synonymous mutations using the dN/dS ratios of the ESL copies of the genus *Arabidopsis* revealed that all these recently diverged genes are subjected to a purifying selection, acting in favor of the sequence conservation at the protein level and, consequently, of their functions as sugar transporters. It is noteworthy that the dN/dS value increases progressively from the group ESL1 (dN/dS = 0.081) through the group ESL2 (dN/dS = 0.172) to the group ESL3 (dN/dS = 0.233), suggesting a slow decrease of the selection pressure. Even though the threshold of one is not reached, we speculate that the progressive increase of dN/dS ratio acts in favor of neutralization or subdiversification toward a possible acquisition of novel functions by ESL MSTs.

As structural changes in protein-coding regions are a phenomenon that can lead to subfunctionalization or neofunctionalization following gene duplication, we analyzed the protein motifs for 421 sequences, including ESL, MST, and SUC as well as the gene structure of the 519 identified *ESL*. We found two conserved protein motifs common to ESL and other sugar transporters and eight motifs conserved with the monosaccharide carriers. The different ESL groups discriminate in the presence or the absence of particular motifs located preferentially in the N-term region. We determined 10 major gene structure types, including 1 specific to *ESL1* of monocots, 1 specific to *ESL2a* of Rutaceae, 1 specific to *ESL2b* of Solanaceae, and 6 specific to Brassicaceae. The first one, named as structure 1, is an 18-exon structure found in more than 50% of the *ESL* sequences, including those from early diverging species. Thus, it seems likely to be an ancestral type that has been preserved throughout evolution. The other 9 17-exon and 16-exon structures have probably appeared in the course of evolution by an intron loss. This hypothesis is in agreement with the literature as it was reported for *Arabidopsis* and rice, that after gene duplication, the rate of intron loss is higher than that of intron gain (Roy and Gilbert, 2005; Lin et al., 2006). However, it is surprising that *ESL1* for monocots and Brassicaceae, which might represent ancient copies, present a 17-exon structure (structures 2 and 4; Figure 9), which implies a more complex evolution. As the exon and intron boundaries play key roles in the evolution of multigene families, the structure of the sugar transporter genes has been analyzed in pears (*Pyrus bretschneideri* Rehd), and it has been demonstrated that the exon numbers of its 75 sugar transporter genes range from 2 to 18 (Li et al., 2015). Our results are in agreement with already demonstrated differences in the structure of different sugar transporter subfamilies, where the PLT, TMT, INT, and STP display more conserved structures with an exon number restricted between one and six, whereas pGlcT, SUT, SFP, and VGT have more exons, ranging from 2 to 18 (Li et al., 2015; Zhang et al., 2021). Taken together our results and data from the literature, it was revealed that exon gain and loss occurring during the evolution of the sugar transporter gene family might lead to the functional diversity of closely related genes.

Although *AtESL* expression levels are variable from gene to gene in *A. thaliana* organs, our expression analysis has shown that the expression of 17 genes could be detected in 5 organs, demonstrating that they are all expressed. On the contrary, *ESL3.09 (At3g05155)* and *ESL3.12 (At3g05165)* could not be amplified nor analyzed as no specific primers could be correctly designed. This is in agreement with the fact that no expression data are available in the Genevestator and BAR databases, suggesting that they are pseudogenes. At least for *ESL3.09 (At3g05155)*, this is corroborated by its short sequence presenting only 13 exons. *ESL1.01* and *ESL1.02* are expressed more or less constitutively in all tested organs and in similar levels. The lack of organ specificity for these *ESL1* genes potentially allows them to respond to a wide range of developmental and/or environmental signals. This is consistent with the hypothesis that *ESL1 gene*s are related to the copies present in the genomes of *P. patens* (moss) and *S. moellendorffii* (fern). *ESL2* genes are rather weakly expressed and only *ESL2.03* shows a preferential expression in roots. In our experimental conditions, *ESL2.01/ZIF2*, is a weakly expressed gene, maybe slightly more expressed in roots, flowers, and siliques. This is in agreement with Remy et al. (2014) who showed a higher expression in roots and flowers. However, they have identified differences in the expression levels of two splice variants, which were not taken into account in our study. For *ESL3* genes, our results highlight a low level of expression for *ESL3.03, ESL3.04*, and *ESL3.1/SFP13* and the fact that *ESL3.08/ERD6* is the most expressed ESL in all tested organs. We demonstrate a preferential expression pattern of *ESL3.08/ERD6* in vegetative organs than in reproductive organs, and conversely, the highest expression of *ESL3.10* in reproductive organs compared to vegetative ones, especially in siliques. The *AtESL3c* genes, whose dN/dS within the genus *Arabidopsis* suggests relaxed negative selection pressure, show different expression patterns in the five tested organs. This suggests a more rapid evolution and some subfunctionalization for these copies. Among the *ESL* tandem pairs, no or weak differences in the expression between the two copies could be detected for the two pairs *ESL1.01/ESL1.02* and *ESL3.05/ESL3.06*. Inversely, a highly significant differential expression is clearly demonstrated for the following tandems: *ESL2.02/ESL3.11* in leaves and siliques*; ESL3.02/ESL3.03* in leaves, flowers, and siliques; *ESL3.13/ESL3.14* in reproductive organs; and *ESL3.07/ESL3.08* in vegetative organs. *ESL3.13/SFP1* shows a weaker expression than *ESL3.14/ SFP2* in all organs and is the weakest expressed *ESL3c*. This result corroborates the Northern blot analysis demonstrating that *ESL3.13/SFP1* is undetectable in young and fully expanded leaves, stems, and roots, whereas *ESL3.14/SFP2* is expressed in these organs (Quirino et al., 2001). *ESL3.13/SFP1* is strongly expressed in seedlings and is highly induced in senescent leaves, whereas *ESL3.14/SFP2* is not (Quirino et al., 2001). *ESL3.08/ERD6* is more expressed than *ESL2.08/ESL1* at least in leaves, roots, and buds. This result is in perfect accordance with the results of Yamada et al. (2010), who also showed that *ESL3.08/ERD6* is more expressed than *ESL2.08/ESL1* in the leaves from the plants growing under normal conditions. They also determined that the transcriptional activity conferred by the promoters of *ESL3.08/ERD6* and *ESL3.07/ESL1* is preferentially located in the same organs, i.e., shoots, sepals, and roots. In roots, *ESL3.08/ERD6* is specifically expressed in epidermal and cortical cells, whereas *ESL3.07/ESL1* is expressed in endoderm, pericycle, and xylemic parenchyma cells (Yamada et al., 2010). The latter data and our own results showing differential and organ-preferential expression suggest possible mechanisms of subfunctionalization for most tandem duplicated *AtESL*.

## Conclusion

The authors performed an evolutionary analysis of plant ESL transporters using the protein sequences from various species representing the main groups of the plant kingdom and demonstrated that these genes arose with streptophytes and have considerably diversified in Embryophytes. Even though the *AtESL* genes are evolving under a purifying selection, their highly variable gene structure and organ-preferential expression emphasize the onset of a plausible diversification. A plethora of promoter *cis*-acting elements tightly related to developmental and environmental cues opens novel perspectives for further elucidation of their biological functions.

## Data Availability Statement

The original contributions presented in the study are included in the article/Supplementary Material, further inquiries can be directed to the corresponding author/s.

## Author Contributions

FD, LS, ML, RA, and RC designed the experiments, discussed the data, and revised the article. AI, CP, FT, and LS performed the experiments. FD, LS, ML, and RA wrote the article. All authors approved the final manuscript.

## Conflict of Interest

The authors declare that the research was conducted in the absence of any commercial or financial relationships that could be construed as a potential conflict of interest.

## Publisher's Note

All claims expressed in this article are solely those of the authors and do not necessarily represent those of their affiliated organizations, or those of the publisher, the editors and the reviewers. Any product that may be evaluated in this article, or claim that may be made by its manufacturer, is not guaranteed or endorsed by the publisher.

## Supplementary Material

Supplementary Figure 1 The linear correlation of the number of tandem duplicated early response to dehydration six-like (ESL). **(A)** The total number of duplicated *ESL* with the total number of *ESL* in each species. **(B)** The total number of duplicated *ESL3* with the total number of *ESL* in each species. **(C)** The total number of eudicots duplicated *ESL3* with the total number of *ESL* in each eudicots species. **(D)** The total number of eudicots duplicated *ESL3c* with the total number of *ESL* in each eudicots species. **(E)** The total number of eudicots duplicated *ESL3c* with the total number of *ESL3* in each eudicots species.

Supplementary Figure 2 Brassicaceae and monocot *ESL* gene structures. Blue rectangles are for exons and lines are for introns. Exons are represented with the same scale and introns with the same length. Red numerals indicate the number of exons.

Supplementary Figure 3 *Cis*-regulatory elements found in the 1-kb promoter of *A. thaliana ESL* genes. **(A)** The distribution of *cis*-elements according to their specificity. **(B)** Common motifs found in all analyzed *ESL* genes.

Supplementary Figure 4 The maximum likelihood phylogeny of monocot ESL sugar transporter proteins and conserved protein motifs. The tree was produced by aligning 67 amino acid ESL sequences identified in 10 species (Spo, Pd, Ph, Pvi, Si, Sb, Bd, Ma, Os, and Zm) using ClustalW and then build using the software Molecular Evolutionary Genetics Analysis (MEGAv10). Jones–Taylor–Thornton (JTT) amino acid substitution model was used and the bootstrap consensus tree was inferred from 1,000 replicates. Motifs were determined by using MEME.

Supplementary Figure 5 The maximum likelihood phylogeny of Brassicales ESL sugar transporter proteins and conserved protein motifs. The tree was produced by aligning the 126 amino acid ESL sequences identified in 7 species (Ah, Al, At, Br, Cp, Cr, and Th) using ClustalW and then build using the software Molecular Evolutionary Genetics Analysis (MEGAv10). JTT amino acid substitution model was used and the bootstrap consensus tree was inferred from 1,000 replicates. Motifs were determined by using MEME.

Supplementary Table 1 Early response to dehydration six-like (ESL) genes identified in this study.

Supplementary Table 2 Primers used for the expression analysis of *AtESL* par real-time reverse transcription-PCR (qRT-PCR).

Supplementary Table 3 Number of ESL genes and tandem duplicated ESL identified in this study.

Supplementary Table 4 Unique *cis*-acting elements identified only in the promoter sequence of a single *Arabidopsis thaliana* ESL transporter gene.

Supplementary Table 5 Protein motifs identified in the sequence of ESL and other sugar transporters proteins. For one motif, each line represents a single analysis performed with a set of sequences indicated in the last column entitled “Analyzed transporters.” The analysis was performed by using an *E*-value < *E*^−60^; however, a few motifs with a higher *E*-value are presented as they are specific to ESL group containing few genes.
