# Supplementary material for: Early Response to Dehydration Six-Like Transporter Family: Early Origin in Streptophytes and Evolution in Land Plants
Source: Front Plant Sci. 2021 Sep 6;12:681929. doi: 10.3389/fpls.2021.681929 (PMC8450595; doi:10.3389/fpls.2021.681929)
Supplement: Supplementary Figure 1 — The linear correlation of the number of tandem duplicated early response to dehydration six-like (ESL). (A) The total number of duplicated ESL with the total number of ESL in each species. (B) The total number of duplicated ESL3 with the total number of ESL in each species. (C) The total number of eudicots duplicated ESL3 with the total number of ESL in each eudicots species. (D) The total number of eudicots duplicated ESL3c with the total number of ESL in each eudicots species. (E) The total number of eudicots duplicated ESL3c with the total number of ESL3 in each eudicots species. [file Data_Sheet_1.zip › Supplementary Figure 2 (2).PPTX]

## Slide 1
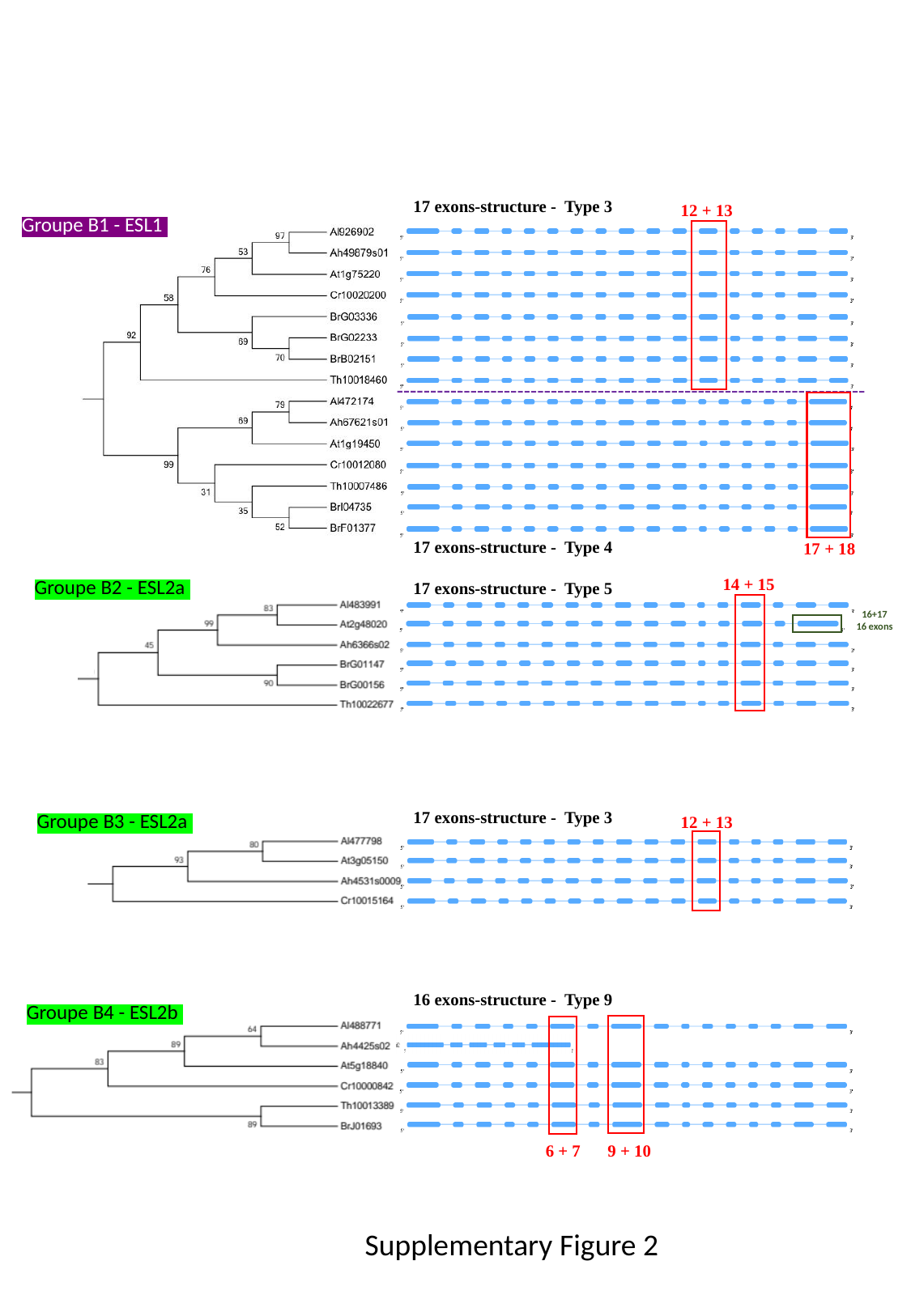

17 exons-structure - Type 3
12 + 13
Groupe B1 - ESL1
17 exons-structure - Type 4
17 + 18
14 + 15
Groupe B2 - ESL2a
17 exons-structure - Type 5
16+17
16 exons
17 exons-structure - Type 3
Groupe B3 - ESL2a
12 + 13
16 exons-structure - Type 9
Groupe B4 - ESL2b
6 + 7
9 + 10
Supplementary Figure 2

## Slide 2
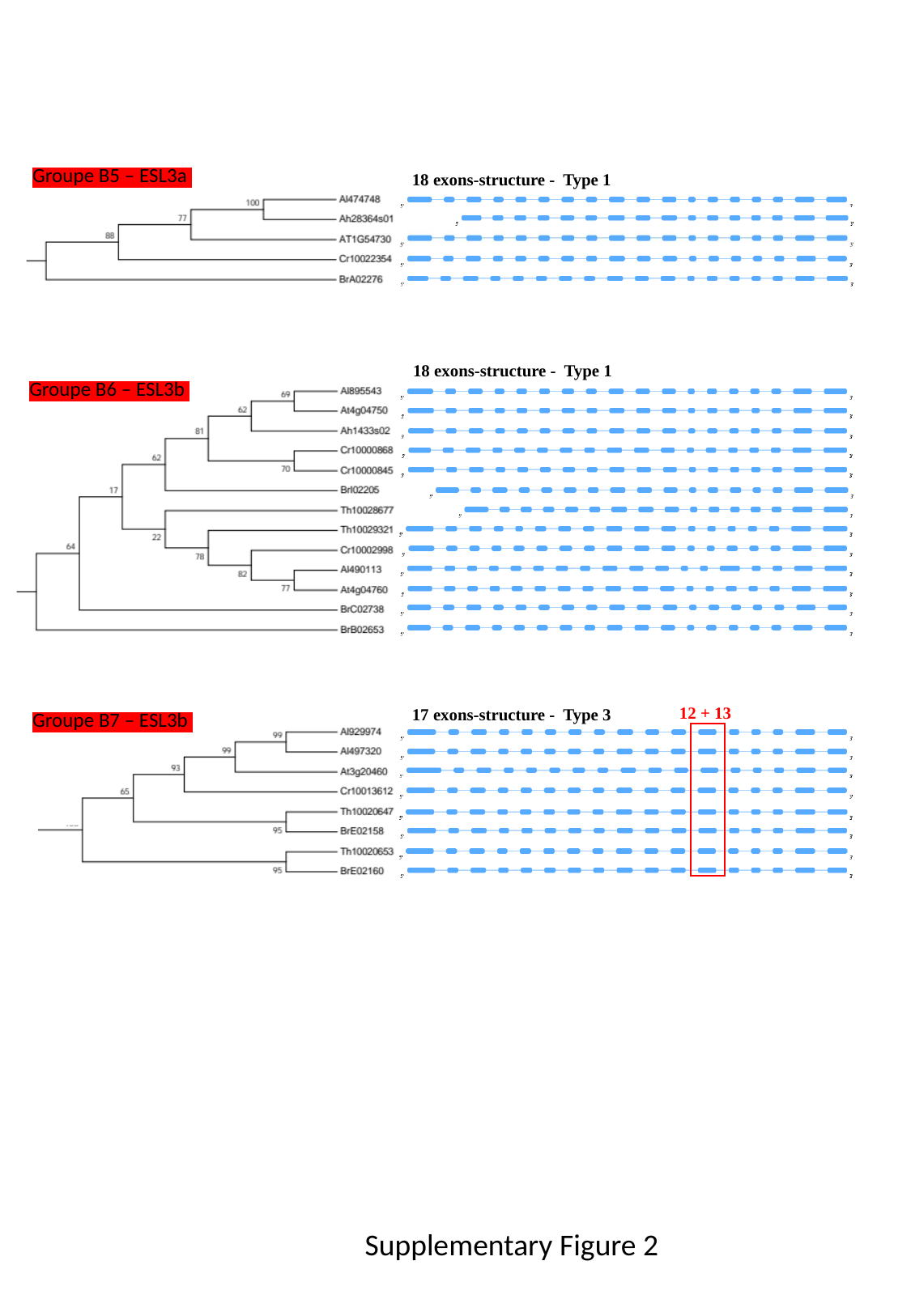

Groupe B5 – ESL3a
18 exons-structure - Type 1
18 exons-structure - Type 1
Groupe B6 – ESL3b
12 + 13
17 exons-structure - Type 3
Groupe B7 – ESL3b
Supplementary Figure 2

## Slide 3
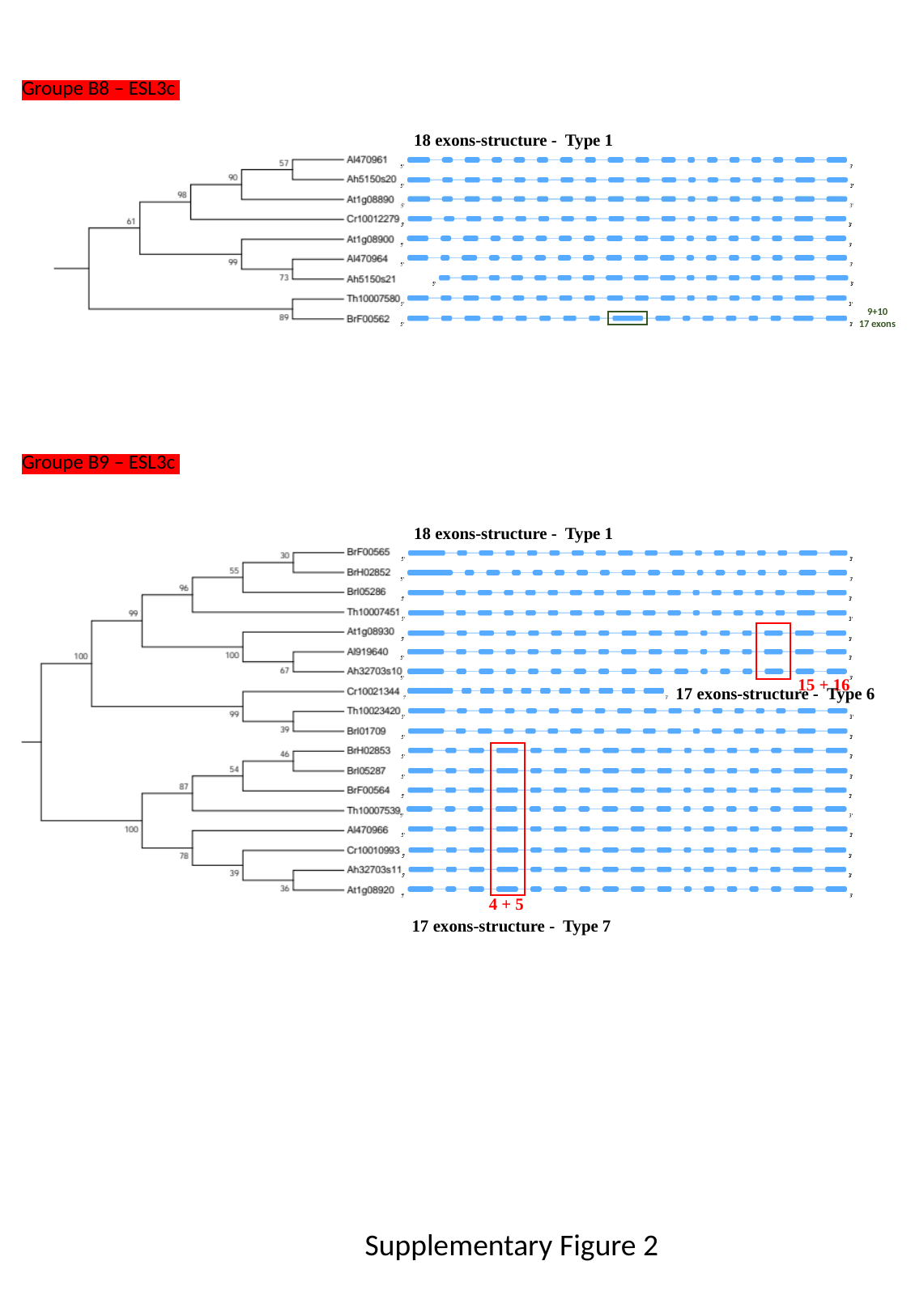

Groupe B8 – ESL3c
18 exons-structure - Type 1
9+10
17 exons
Groupe B9 – ESL3c
18 exons-structure - Type 1
15 + 16
17 exons-structure - Type 6
4 + 5
17 exons-structure - Type 7
Supplementary Figure 2

## Slide 4
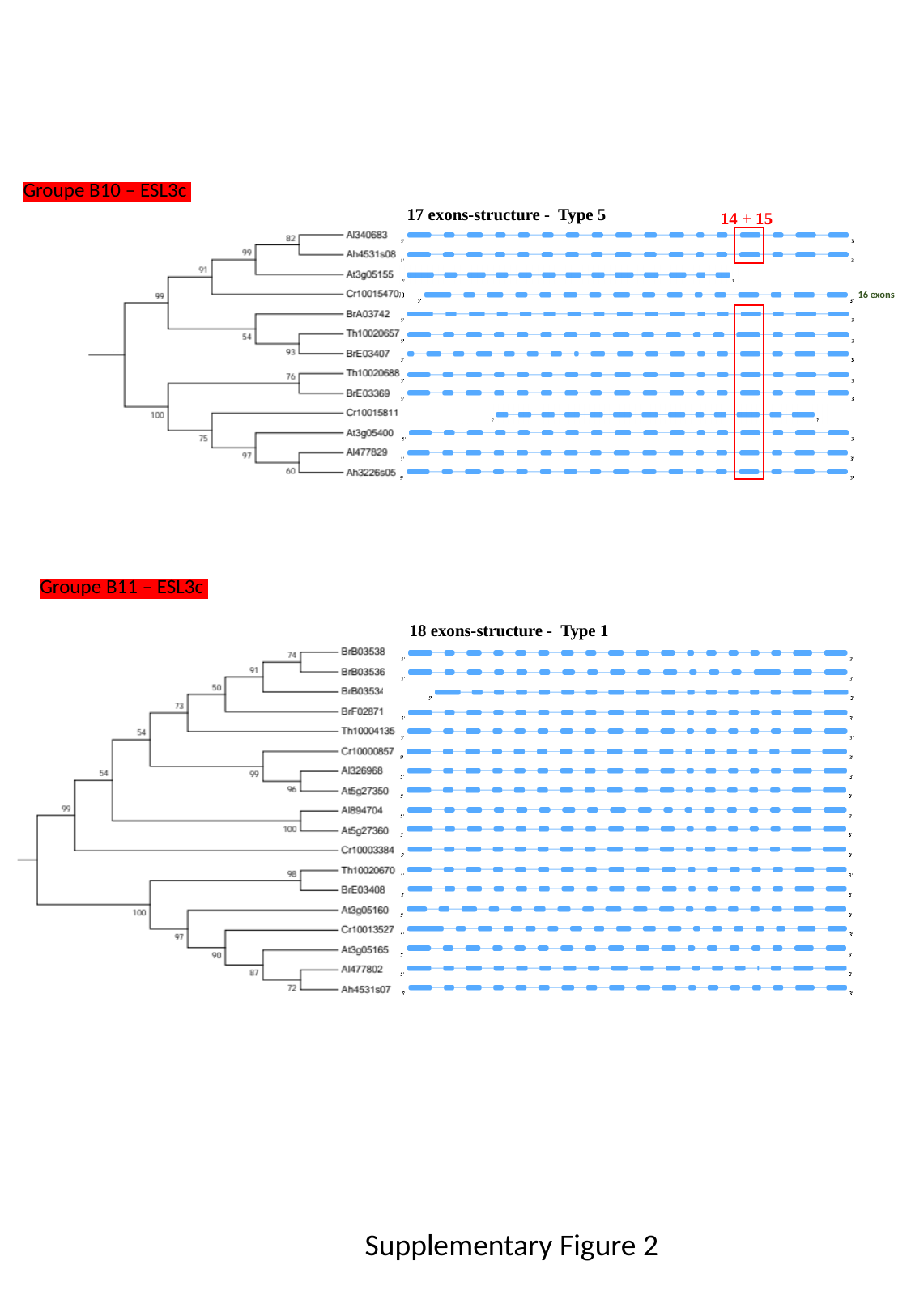

Groupe B10 – ESL3c
17 exons-structure - Type 5
14 + 15
16 exons
Groupe B11 – ESL3c
18 exons-structure - Type 1
Supplementary Figure 2

## Slide 5
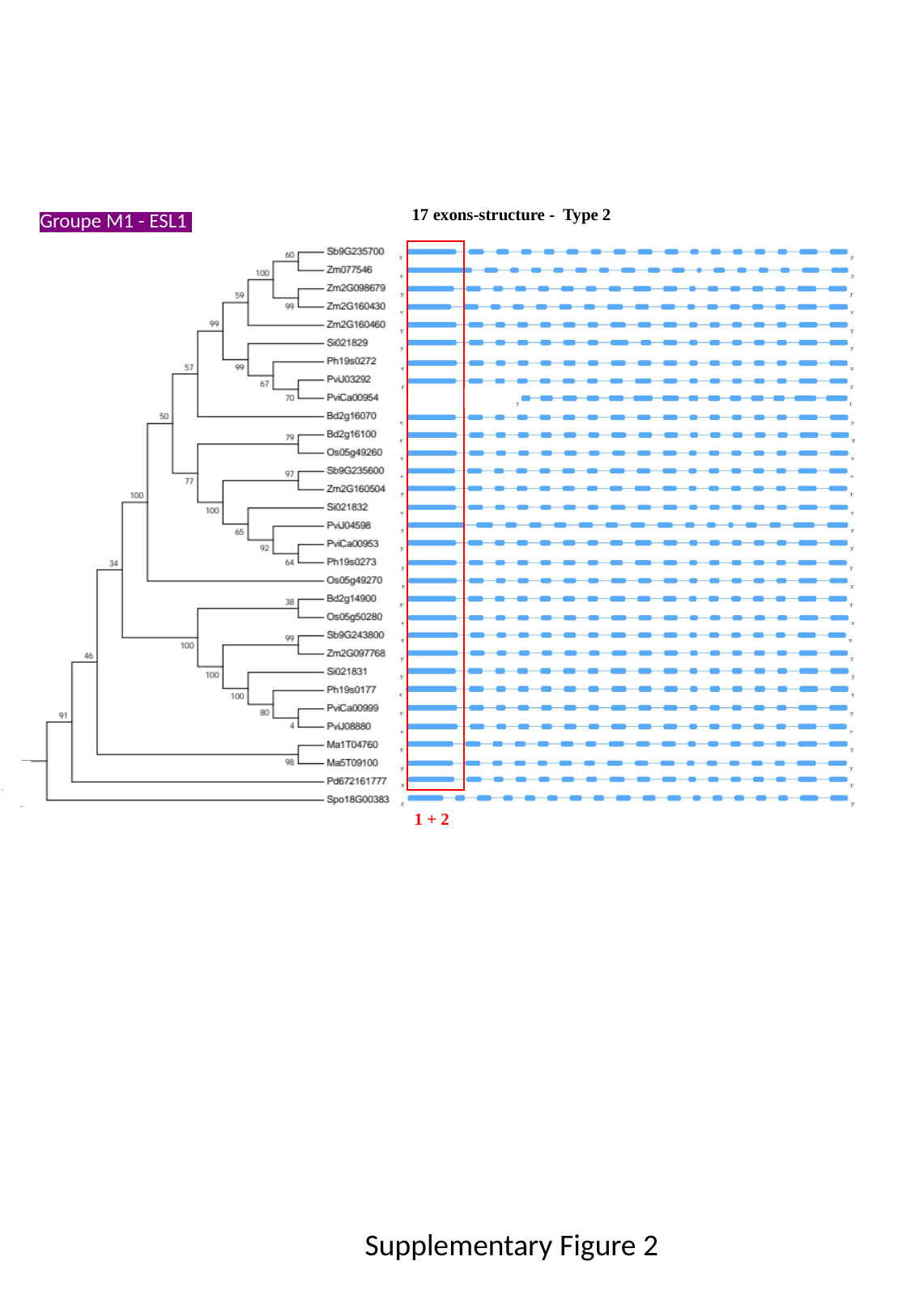

17 exons-structure - Type 2
Groupe M1 - ESL1
1 + 2
Supplementary Figure 2

## Slide 6
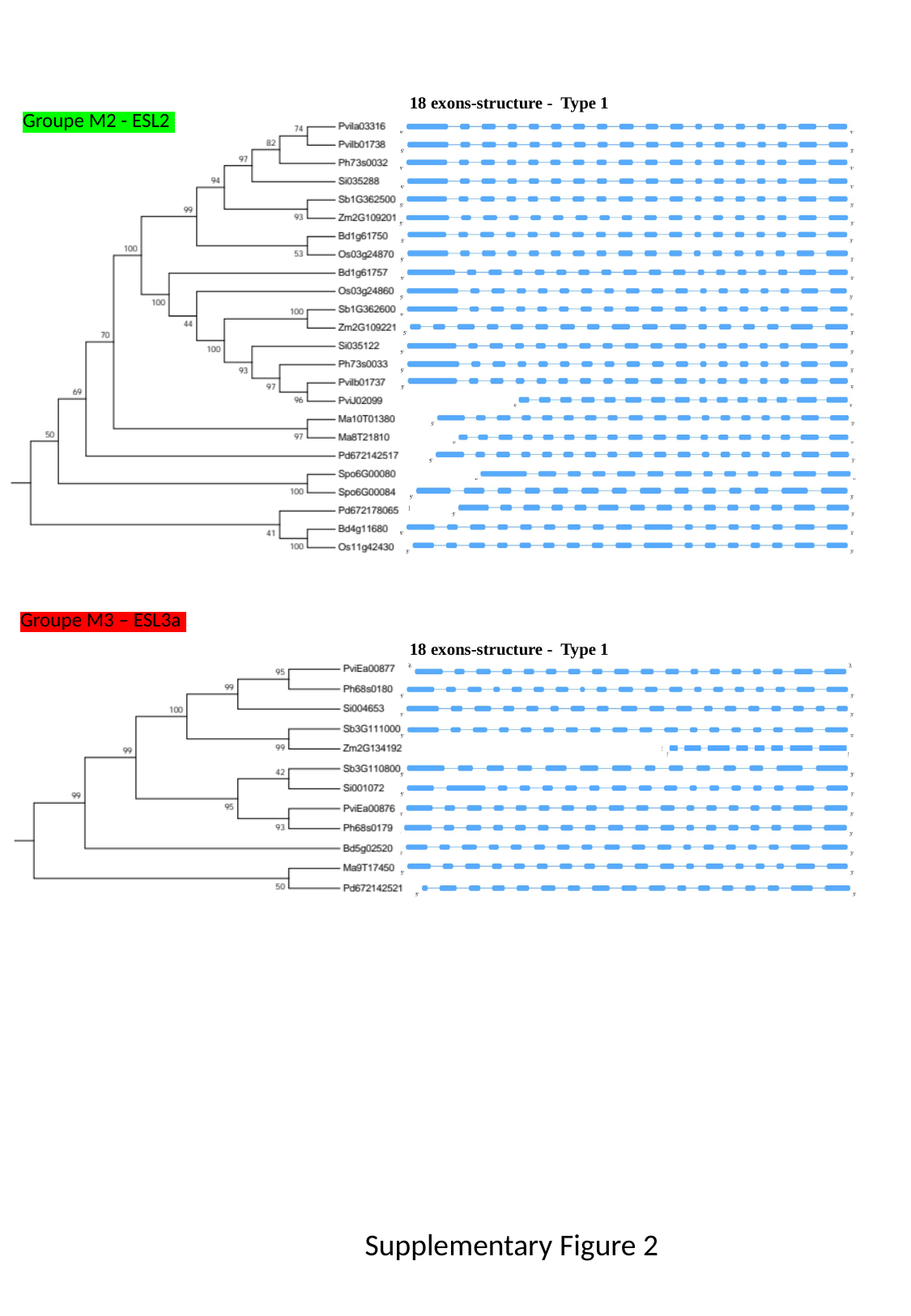

18 exons-structure - Type 1
Groupe M2 - ESL2
Groupe M3 – ESL3a
18 exons-structure - Type 1
Supplementary Figure 2
