# Supplementary figures and images for: Early Response to Dehydration Six-Like Transporter Family: Early Origin in Streptophytes and Evolution in Land Plants
Source: Front Plant Sci. 2021 Sep 6;12:681929. doi: 10.3389/fpls.2021.681929 (PMC8450595; doi:10.3389/fpls.2021.681929)

## Slide 1
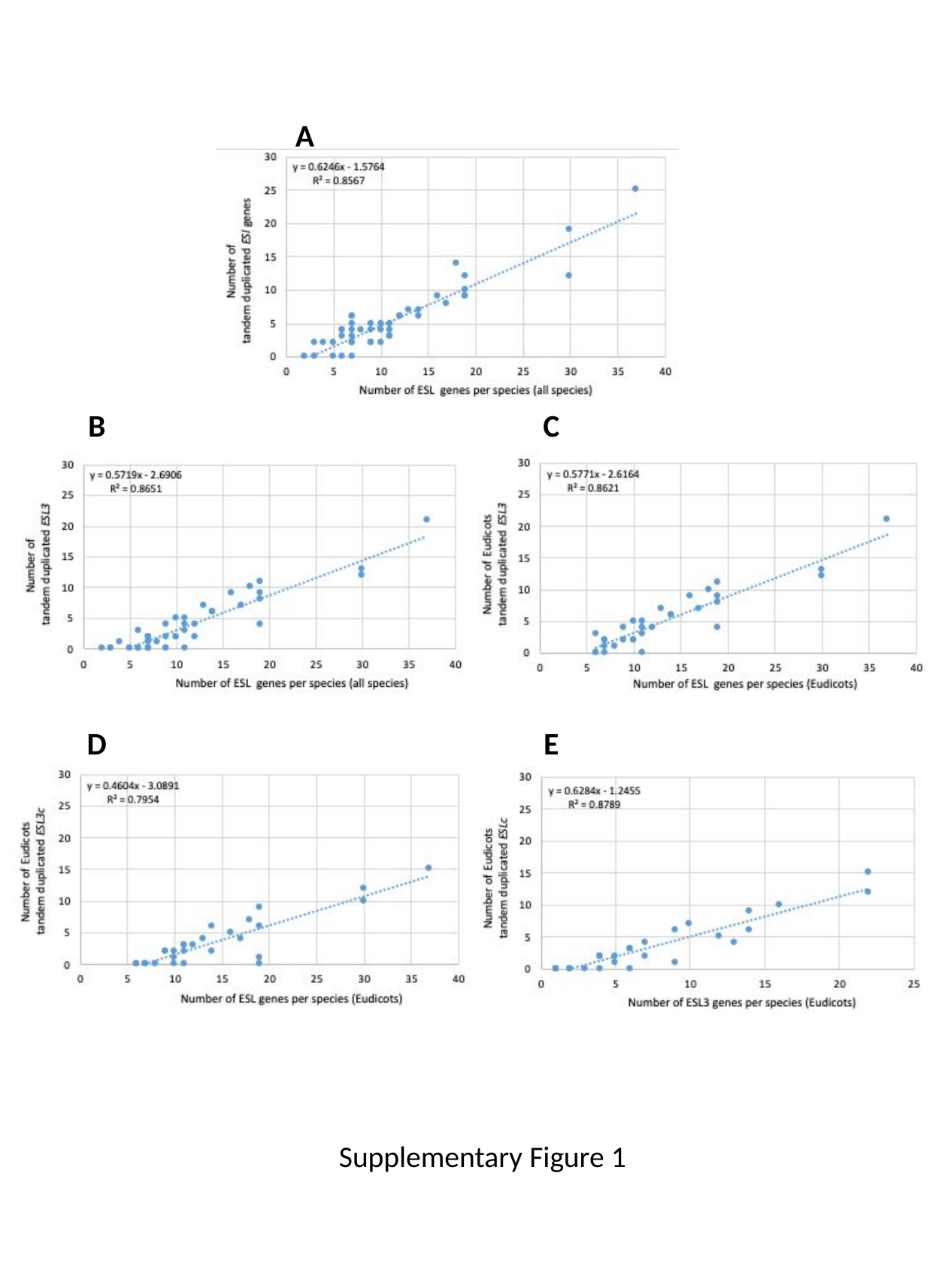

A
B
C
D
E
Supplementary Figure 1

Supplement: Supplementary Figure 1 — The linear correlation of the number of tandem duplicated early response to dehydration six-like (ESL). (A) The total number of duplicated ESL with the total number of ESL in each species. (B) The total number of duplicated ESL3 with the total number of ESL in each species. (C) The total number of eudicots duplicated ESL3 with the total number of ESL in each eudicots species. (D) The total number of eudicots duplicated ESL3c with the total number of ESL in each eudicots species. (E) The total number of eudicots duplicated ESL3c with the total number of ESL3 in each eudicots species. [file Data_Sheet_1.zip › Supplementary Figure 1 (1).PPTX]

## Slide 1
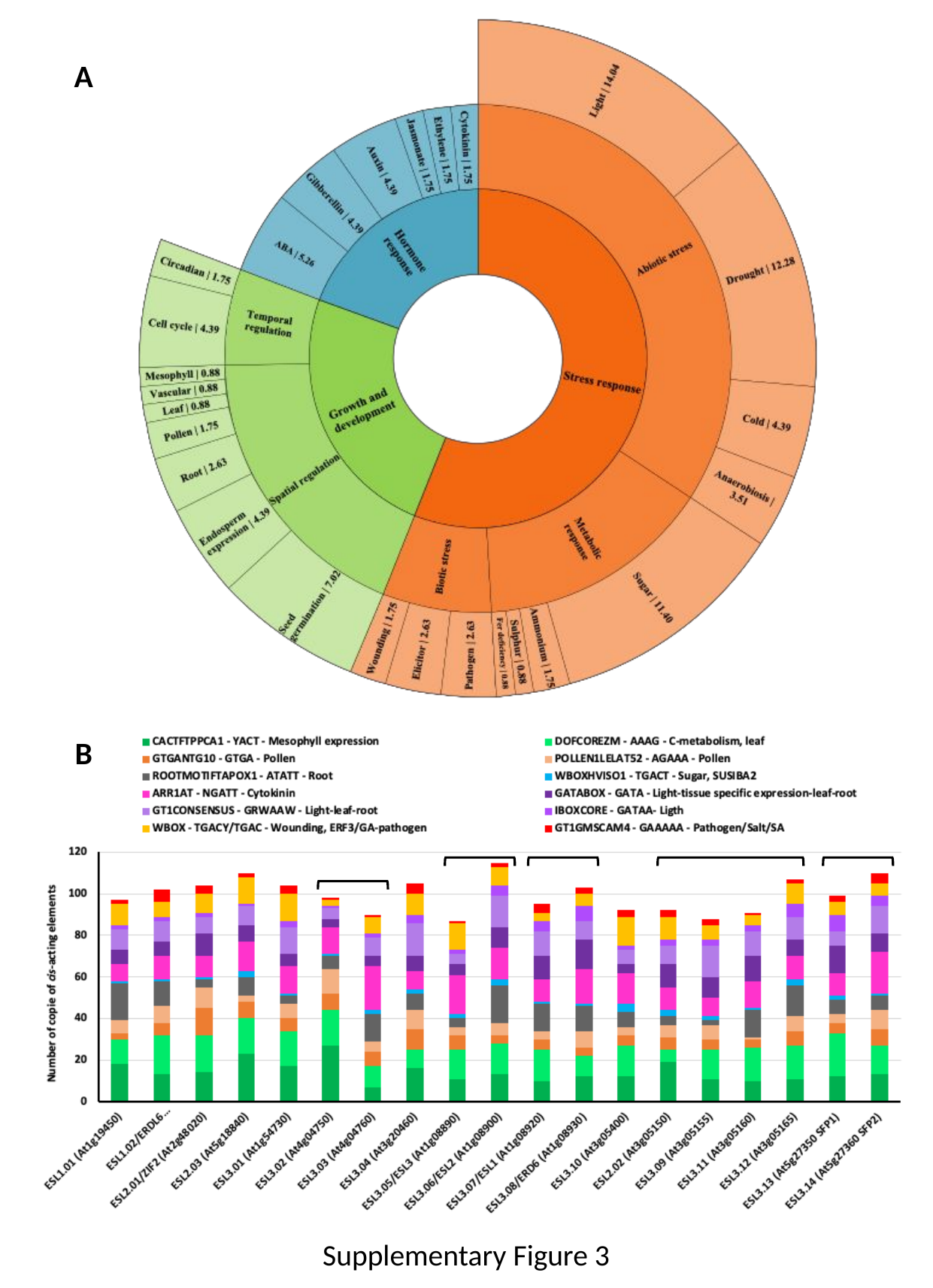

A
B
Supplementary Figure 3

Supplement: Supplementary Figure 1 — The linear correlation of the number of tandem duplicated early response to dehydration six-like (ESL). (A) The total number of duplicated ESL with the total number of ESL in each species. (B) The total number of duplicated ESL3 with the total number of ESL in each species. (C) The total number of eudicots duplicated ESL3 with the total number of ESL in each eudicots species. (D) The total number of eudicots duplicated ESL3c with the total number of ESL in each eudicots species. (E) The total number of eudicots duplicated ESL3c with the total number of ESL3 in each eudicots species. [file Data_Sheet_1.zip › Supplementary Figure 3.PPTX]

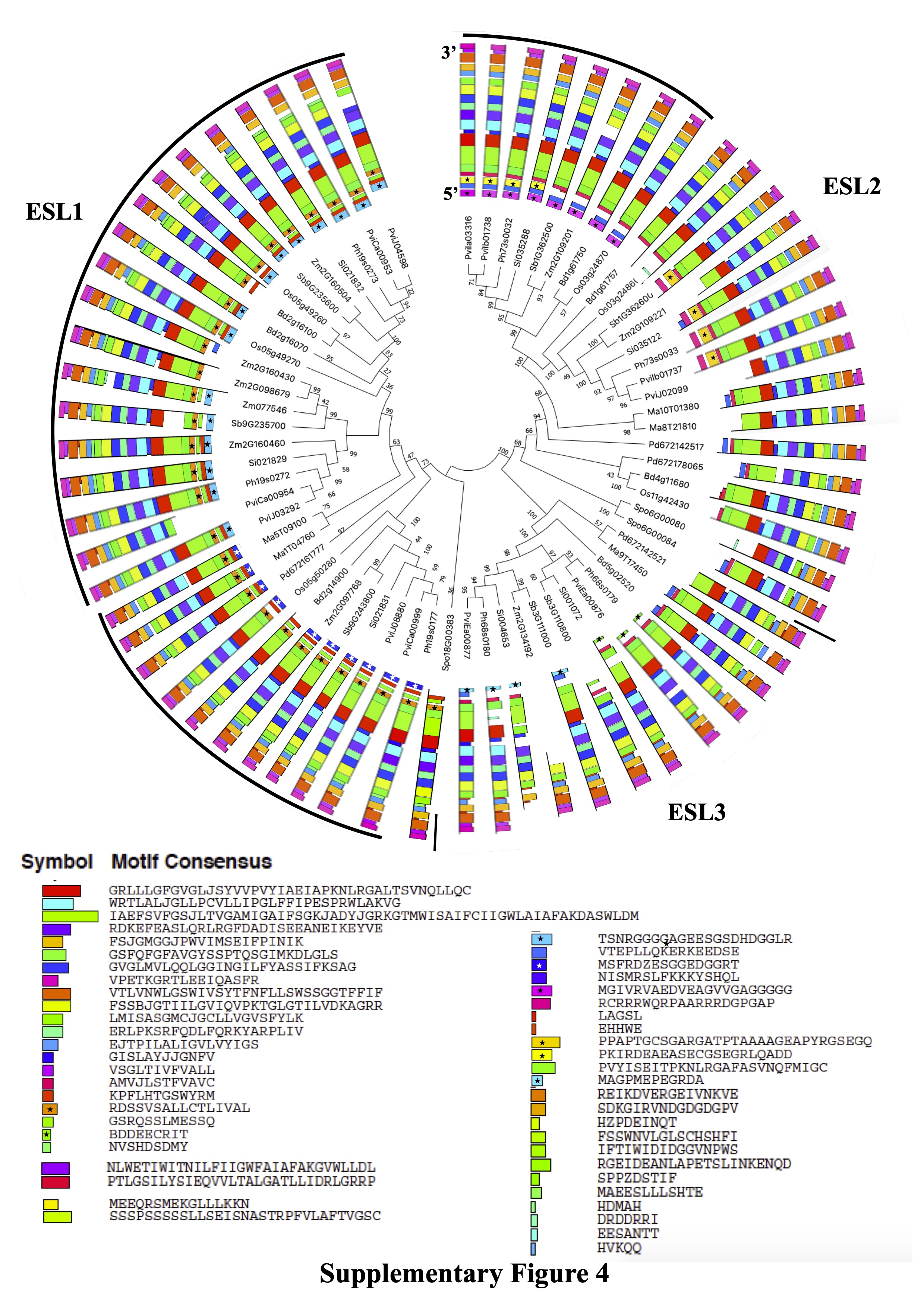

Supplement: Supplementary Figure 1 — The linear correlation of the number of tandem duplicated early response to dehydration six-like (ESL). (A) The total number of duplicated ESL with the total number of ESL in each species. (B) The total number of duplicated ESL3 with the total number of ESL in each species. (C) The total number of eudicots duplicated ESL3 with the total number of ESL in each eudicots species. (D) The total number of eudicots duplicated ESL3c with the total number of ESL in each eudicots species. (E) The total number of eudicots duplicated ESL3c with the total number of ESL3 in each eudicots species. [file Data_Sheet_1.zip › Supplementary Figure 4.JPEG]

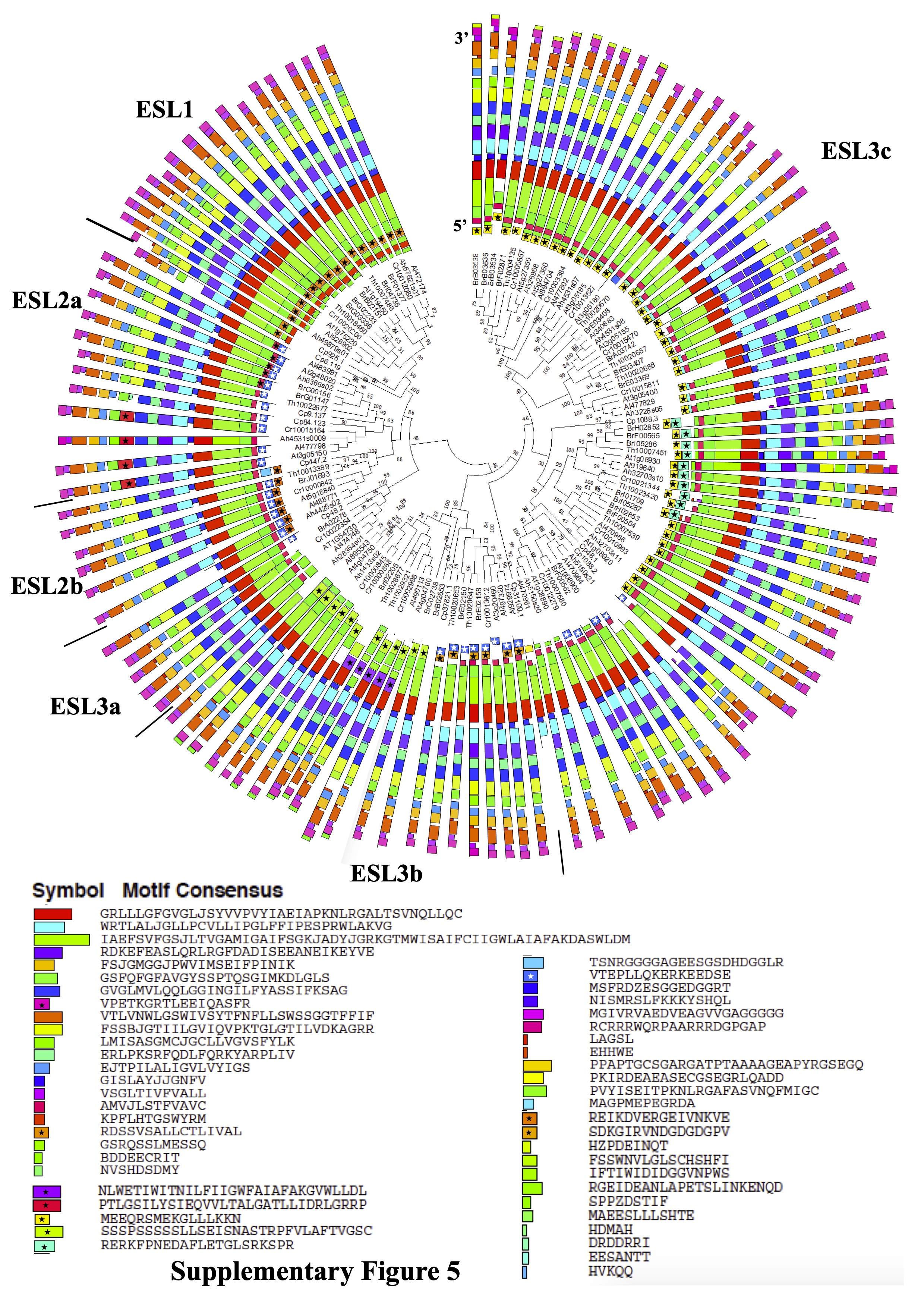

Supplement: Supplementary Figure 1 — The linear correlation of the number of tandem duplicated early response to dehydration six-like (ESL). (A) The total number of duplicated ESL with the total number of ESL in each species. (B) The total number of duplicated ESL3 with the total number of ESL in each species. (C) The total number of eudicots duplicated ESL3 with the total number of ESL in each eudicots species. (D) The total number of eudicots duplicated ESL3c with the total number of ESL in each eudicots species. (E) The total number of eudicots duplicated ESL3c with the total number of ESL3 in each eudicots species. [file Data_Sheet_1.zip › Supplementary Figure 5.JPEG]
