# Supplementary material for: Early Response to Dehydration Six-Like Transporter Family: Early Origin in Streptophytes and Evolution in Land Plants
Source: Front Plant Sci. 2021 Sep 6;12:681929. doi: 10.3389/fpls.2021.681929 (PMC8450595; doi:10.3389/fpls.2021.681929)
Supplement: Supplementary Table 1 — Early response to dehydration six-like (ESL) genes identified in this study. [file Data_Sheet_2.zip › Supplementary Table 2.pdf]

SUPPLEMENTARY Table 2 :
Primers used for the expression analysis of *AtESL* par qRT-PCR.

Genes	Sequences	Tm (°C)	Length (nt)	Taille de l'amplicon (bp)	Efficacité	Observations
At1g19450	F : TAAGCCTCGTGATTGTAGCA	58	20	121	90	-
	R : CAAGAAATAACCATAGCCACA	58	21			
At1g75220	F : GTGGAGGAACTTTCACTCTGTA	64	22	99	90	-
	R : GTTCTTCAAGAGTTTTGCCTT	58	21			
At2g48020	F : CTGAGATATTTCCCATAAACAT	58	21	139	93	-
	R : AAGATTGCAATTTATGTCCCAG	58	21			
At3g05150	F : TTCTTTTGAAGGCTCATGGTT	58	21	200	-	-
	R : GACAAGCCATGAACTTAGCCA	62	19			
At5g18840	F : CTGAGATATTTCCGATAAACGTA	62	23	177	114	-
	R : TCTTCGCCACAAATATTATCGTC	64	23			
At1g54730	F : AATCCGGCAGGAACGTTTTATG	64	22	179	100	-
	R : CGTTATAATTAGGGACTGATAT	58	22			
At4g04750	F : TACAGGAACGTTTATGATGTTT	58	22	203	90	-
	R : CATGAAATATAAAGAGATCTGA	56	22			
At4g04760	F : CAGAGATTTATCCAGTAGATGT	60	22	174	100	-
	R : TGGCTATGAACACAAATCCAA	58	21			
At3g05400	F : CTTCTTATGACTTCAGCGTTGG	64	22	148	100	-
	R : CAATTGCGAACATCATCGT	54	19			
At1g08890	F : TGGAGTGCTTTCGGAACATATT	60	22	150	109	-
	R : GAGTAAAGAATCAACTTTGGAT	58	22			
At1g08900	F : GTAGCCTTGTAACGATGTCA	58	20	99	97	-
	R : ATATGAAATATGTTCCGGAAGC	60	22			
At3g20460	F: ACGCTATGCAATTTAACTAGCTG	64	23	89	97	-
	R : AACACACCAGAAGAGCTCCACT	66	22			
At1g08920	F : TCGGTCTCATTCCATGTGCG	62	20	131	97	-
	R : TGCATCATCTCCGCGAAGAC	62	20			
At1g08930	F : ATAATGGCTGAGATATTTCCG	58	21	176	95	-
	R : ATAAATACGATCGAACTGGC	60	20			
At3g05155	F : GTGGTACCAAAAGCTATATTGG	62	22	164	-	No amplification
	R : CCGATGCCTGCAAATTTGTACA	64	22			
At3g05160	F : GCACAAGGAACCTTTTACATAT	60	22	101	90	-
	R : AATTCTTCAAGCGACTGTCC	58	20			
At3g05165	F : AAGGAACATTTTACATATTTGC	56	22	167	-	Not enougth specificity
	R : TTTATTATTTGCAATGCGACT	54	21			
At5g27350	F : TATGTCGGAGATATTTCCAATA	58	22	269	97	-
	R : CAAATTAAGTTTGATTTCTTTC	54	22			
At5g27360	F : AGTGGAGCACTCAAGGAACATT	64	22	149	95	-
	R : TTGATTTATTCTATCGGGCTC	60	22			
